# Cytotoxicity and oxidative stress induced by atmospheric mono-nitrophenols in human lung cells

**DOI:** 10.1016/j.envpol.2022.119010

**Published:** 2022-02-22

**Authors:** Faria Khan, Mohammed Jaoui, Krzysztof Rudziński, Karina Kwapiszewska, Alicia Martinez-Romero, Domingo Gil-Casanova, Michael Lewandowski, Tadeusz E. Kleindienst, John H. Offenberg, Jonathan D. Krug, Jason D. Surratt, Rafal Szmigielski

**Affiliations:** a Institute of Physical Chemistry, Polish Academy of Sciences, Kasprzaka 44/52, 01-224, Warsaw, Poland; b Center for Environmental Measurement & Modeling, U.S. Environmental Protection Agency, Research Triangle Park, NC, 27711, United States; c Cytomics Core Facility, Príncipe Felipe Research Center, Avda. Eduardo Primo Yúfera, 3, 46012, Valenica, Spain; d Department of Environmental Sciences and Engineering, Gillings School of Global Public Health, University of North Carolina at Chapel Hill, Chapel Hill, NC, 27599, United States; e Department of Chemistry, University of North Carolina at Chapel Hill, Chapel Hill, NC, 27599, United States

**Keywords:** Ambient aerosol, Aromatic hydrocarbons, Inhibitory Concentration-50, Reactive oxygen species, BEAS-2B cells, A549 cells

## Abstract

Nitrophenols (NPs) are hazardous pollutants found in various environmental matrices, including ambient fine particulate matter (PM_2.5_), agricultural residues, rainwater, wildfires, and industrial wastes. This study showed for the first time the effect of three pure nitrophenols and their mixture on human lung cells to provide basic understanding of the NP influence on cell elements and processes. We identified NPs in ambient PM_2.5_ and secondary organic aerosol (SOA) particles generated from the photooxidation of monocyclic aromatic hydrocarbons in the U.S. EPA smog chamber. We assessed the toxicity of identified NPs and their equimolar mixture in normal bronchial epithelial (BEAS-2B) and alveolar epithelial cancer (A549) lung cell lines. The inhibitory concentration-50 (IC_50_) values were highest and lowest in BEAS-2B cells treated with 2-nitrophenol (2NP) and 4-nitrophenol (4NP), respectively, at 24 h of exposure. The lactate dehydrogenase (LDH) assay showed that 4NP, the most abundant NP we identified in PM_2.5_, was the most cytotoxic NP examined in both cell lines. The annexin-V/fluorescein isothiocyanate (FITC) analysis showed that the populations of late apoptotic/necrotic BEAS-2B and A549 cells exposed to 3NP, 4NP, and NP equimolar mixture increased between 24 and 48 h. Cellular reactive oxygen species (ROS) buildup led to cellular death post exposure to 3NP, 4NP and the NP mixtures, while 2NP induced the lowest ROS buildup. An increased mitochondrial ROS signal following NP exposure occurred only in BEAS-2B cells. The tetramethylrhodamine, methyl ester, perchlorate (TMRM) assay showed that exposed cells exhibited collapse of the mitochondrial membrane potential. TMRM signals decreased significantly only in BEAS-2B cells, and most strongly with 4NP exposures. Our results suggest that acute atmospheric exposures to NPs may be toxic at high concentrations, but not at ambient PM_2.5_ concentrations. Further chronic studies with NP and NP-containing PM_2.5_ are warranted to assess their contribution to lung pathologies.

## 1. Introduction

2-Nitrophenol (2NP), 3-nitrophenol (3NP), and 4-nitrophenol (4NP) are aromatic compounds with one benzene ring, one OH group at position 1, and one NO_2_ group at positions 2 (*ortho*), 3 (*meta*), and 4 (*para*), respectively. They originate from the combustion of vehicle fuels (Claeys et al., 2012; Iinuma et al., 2010; Inomata et al., 2015; Lin et al., 2016; Lin et al., 2017a; Lu et al., 2019a; Rubio et al., 2019), biomass burning and wildfires (Bluvshtein et al., 2017; Laskin et al., 2015; Lin et al., 2017a; Xie et al., 2019a), incineration plants (Jay and Stieglitz, 1995), degradation of pesticides (Boehncke et al., 2000; EPA, 2006; WHO, 2004), industrial processes (Boehncke et al., 2000; Harrison et al., 2005), and atmospheric oxidation of aromatic compounds in the presence of nitrogen oxides (NO_x_) (Al-Naiema and Stone, 2017; Harrison et al., 2005; Lin et al., 2015; Vione et al., 2005; Vione et al., 2006; Xie et al., 2017). Nitrophenols (NPs) occur worldwide in water, fog, rain, clouds, soil, snow, and ambient fine particulate matter (PM_2.5_, aerosol particles with aerodynamic diameters *<*2.5 μm) (Cecinato et al., 2005; Harrison et al., 2005; Xie et al., 2019b).

Emission and atmospheric behavior of NPs are priority topics in atmospheric research as they impact the Earth’s climate by the formation of light-absorbing PM_2.5_ (Bluvshtein et al., 2017; Chow et al., 2016; Li et al., 2020; Lin et al., 2016; Lin et al., 2017a; Lu et al., 2019b; Xie et al., 2019b; Xie et al., 2017), and through inhalation of NP-containing PM_2.5_ could have adverse effects on human health (Gramatica et al., 2002). 2NP and 4NP are priority hazardous pollutants (EPA, 2014a, b; Keith and Telliard, 1979). Mono- and di-NPs were toxic in plants and mammals (Gramatica et al., 2002), while 4NP was highly toxic in humans (Schafer et al., 2004).

The *in-vitro* and *in-vivo* NPs studies are scarce, and only a few have examined the oral exposures to NPs (Boehncke et al., 2000; Vernot et al., 1977). The 2NP oral LD_50_ (lethal dose, 50%) was 2830–5376 mg kg^−1^ body weight (bw) in rats and 1300–2080 mg kg^−1^ bw in mice. The 3NP LD_50_ was 930 mg kg^−1^ bw in rats and 1070 mg kg^−1^ bw in mice. The 4NP LD_50_ was 220–620 mg kg^−1^ bw in rats (Boehncke et al., 2000; Vernot et al., 1977). 4NP and 2NP were not considered carcinogenic (EPA, 2006), and mutagenicity of 2NP, 3NP, and 4NP was not declared (Boehncke et al., 2000). A recent *in-vitro* experiment showed that 2NP destroyed DNA (Zhang et al., 2008), while a genotoxic risk assessment of 3NP as a drug impurity showed that a 4 mg day^−1^ threshold was safe for the intra-dermal application (Eichenbaum et al., 2009).

Transfer of NPs into lung fluids during inhalation of NP-containing PM_2.5_ is probable in industrial and urban settings, as well as in regions with high biomass burning emissions (Boehncke et al., 2000; Harrison et al., 2005). NPs are sufficiently soluble in water to warrant partitioning from the inhaled gas phase and particles into lung fluids (Information, 2021a, b, c; Yalkowsky et al., 2001). However, water solubility can roughly estimate the dissolution of NPs in lung fluids that are not pure water. Thus, assessing bio-accessibility of NPs during inhalation requires that the mass transfer parameters determined for solutions are similar to lung fluids (Calas et al., 2017). For instance, Kitanovski et al. (2020a) found that artificial lung fluid (pH = 4.5) leached on average 78% of 4NP contained within PM_2.5_ collected at the Kladno and Ostrava sites (Czech Republic), and Gamble’s Solution (pH = 7.4) leached 64% of 4NP from the same PM_2.5_ samples.

A549 and BEAS-2B lung cell lines have been used for *in vitro* assessment of oxidative stress and cytotoxicity following exposure to PM_2.5_ and gas-phase pollutants (Arashiro et al., 2016; Jathar et al., 2020; Khan et al., 2021; Lima de Albuquerque et al., 2021; Lin et al., 2017b; McDonald et al., 2010; Zhang et al., 2017). Reactive oxygen species (ROS) buildup in exposed cells demonstrates pathophysiological lung conditions (Qu et al., 2017; Zhang et al., 2017). Exposures of lung cells to black carbon and nitro-compounds induced cellular death (apoptosis) (Jiang et al., 2020; Øya et al., 2011). Chronic exposures to nitro-compounds, including NPs, complicated the pre-existing cardio--pulmonary pathologies (allergy, asthma, and respiratory infections) and induced premature death, likely through ROS buildup and apoptosis induction (Janssen Nicole et al., 2011; Pan et al., 2018; Zhang et al., 2017). Mitochondrial dysfunction links to underlying lung pathologies, respiratory diseases, and apoptosis induced by mitochondrial-ROS (mtROS) (Cloonan and Choi, 2016; Liu et al., 2020; Piantadosi and Suliman, 2017). The mtROS disturbance could signal mitochondrial dysfunction after exposure to pollutants (Liu and Chen, 2017).

Equimolar mixtures of 2NP, 3NP, and 4NP caused changes in the eukaryotic cell membrane potential at concentrations above 100 μg mL^−1^ (Majewska et al., 2021). Electrochemical impedance spectroscopy and atomic force microscopy showed that exposure to NP mixtures profoundly rearranged model eukaryotic cell membrane structures, let NPs internalize, and changed cellular morphology. The uptake of the cellular NPs evidenced an intrinsic cellular death mechanism (Cao et al., 2021).

This study aimed to research for the first time the effect of three pure nitophenols and their mixture on human lung cells to throw light on the mechanism of NP interaction with cell elements and cellular processes. We qualitatively identified three NPs in ambient PM_2.5_ collected at several U.S. sites and in SOA particles formed by the photooxidation of aromatic hydrocarbons (benzene, toluene, *o*-xylene, *m*-xylene, 1,3,5-trimethylbenzene, 1,2,4-trimethylbenzene, ethylbenzene, naphthalene, 1-methylnaphthalene, 2-methylnaphthalene, and benzyl alcohol) in the U. S. EPA smog chambers in the presence of NO_x_. We carried out a detailed *in-vitro* toxicological assessment to estimate the uptake effect of 2NP, 3NP, 4NP, and their mixture in lung cell models and explain the intracellular mechanisms following exposure. The latter included the mechanism of cellular death and the underlying ROS buildup, focusing on mitochondrial dysfunction and mtROS. Our study highlighted the early biological changes in the exposed lung cells.

## 2. Materials and methods

### 2.1. Chemicals and probes

We used: 2NP, 3NP, and 4NP (99% purity grade), DMSO (Molecular Biology Grade), and Dulbecco’s phosphate-buffered saline (PBS) from Sigma Aldrich (Merck), Poland; high-purity chemicals for gas chromatography/mass spectrometry (GC-MS) from Aldrich Chemical Co. (Milwaukee), USA, used without further purification; solvents for GC-MS (GC^2^ quality) analysis from Burdick and Jackson (Muskegon, MI), USA; and Milli-Q water (18.2 MΩ) from a Millipore Advantage system (Merck), Poland.

We used: Pierce™ lactate dehydrogenase (LDH) cytotoxicity assay kit, Vybrant MTT assay kit (3-(4,5-dimethylthiazol-2-yl)-2,5-diphenyltetrazolium bromide, calcein-AM, propidium iodide (PI), carboxy-dihydro dichlorofluorescein diacetate (carboxy-H_2_DCFDA), MitoSOX™ red, Hoechst 33342 solution, tetramethylrhodamine methyl ester (TMRM), DAPI (4′,6-diamidino-2-phenylindole), and live-cell imaging solution from Invitrogen (ThermoFisher Scientific, USA); HEPES (Bioultra for Molecular Biology Grade) from Merck;, USA Annexin V-FITC kit from Miltenyi Biotec, Spain; and Trypan blue solution and Triton X-100 solutions from Merck, Poland.

### 2.2. Collection and analysis of Mono-NPs in SOA and ambient PM_2.5_

Experiments were conducted during the past 20 years in a U.S. EPA smog chamber (Docherty et al., 2018; Offenberg et al., 2006) by irradiating individual aromatic hydrocarbons in the presence of NO_x_. The chamber worked either in static mode (as batch reactor) or in dynamic flow mode (as a continuous stirred reactor), at steady-state concentrations of gas- and particle-phase reaction products (Jaoui et al., 2014). See Offenberg et al. (2006) and Docherty et al. (2018) for exact details on chamber operations, procedures, and instruments. Gas-phase organic species were collected in 60 cm, four-channel XAD4-coated annular denuders, and SOA particles onto 47-mm glass-fiber filters (Jaoui et al., 2014). We washed organic compounds from denuders with a 1:1 dichloromethane/methanol mixture. We derivatized the washed compounds with a N,O-bis-(trimethylsilyl)trifluoroacetamide/1% trimethylchlorosilane (BSTFA/TMCS) mixture (Jaoui et al., 2004). The SOA particle samples were extracted by sonication in methanol, dried, and derivatized with a BSTFA/TMCS mixture, and analyzed by GC-MS on a ThermoQuest (Austin, TX) GC coupled with an ion-trap mass spectrometer (Jaoui et al., 2014). The temperature of the injector was 270 °C, and the GC operated in spitless mode. A 60m, 0.25 mm inner diameter, RTx-5MS column (Restek, Inc., Bellefonte, PA) with a 0.25-μm film coating was used. The initial oven temperature was 84 °C for 1 min, increased by a ramp of 8 °C min^−1^ to 200 °C, followed by a 2min hold, and increased by the second ramp of 10 °C min^−1^ to 300 °C. The ion source, ion trap, and interface temperatures were 200, 200, and 300 °C, respectively. 2 μL of the extract was injected in CI and/or EI modes.

Ambient PM_2.5_ samples were collected at several sites in the U.S. between 2003 and 2010 (Table S2) as previously described (Jaoui et al., 2019; Kleindienst et al., 2007; Lewandowski et al., 2013). Briefly, PM_2.5_ samples were collected onto pre-baked quartz-fiber filters for 24 h. Immediately after collection, the filters were spiked with known amounts of ketopinic acid and d_50_-tetracosane as internal and recovery standards, respectively, and Soxhlet extracted for 24 h in a 50-mL dichloromethane/methanol mixture (1:1 v/v). Each extract was evaporated to dryness with nitrogen at room temperature, then derivatized for 1 h with 200 μL of BSTFA/TMCS mixture and 100 μL of pyridine at 70 °C. Derivatized PM_2.5_ sample extracts were analyzed exactly like chamber-generated SOA particle samples.

### 2.3. MTT assay and inhibitory Concentration-50 (IC_50_)

Details of cell culture and medium are provided in the supplementary section S1.1. We used Vybrant MTT assay kit to calculate the percentage cellular proliferation following exposure to aqueous solutions of 2NP, 3NP, 4NP, or their equimolar mixture (0.01–200 μg mL^−1^), for 24 or 48h. For details of the MTT assay protocol, see (Khan et al., 2021). Briefly, cells were seeded in 96-well plates (~10,000 BEAS-2B cells well^−1^ and ~8000 A549 cells well^−1^). After 16h, cells were washed with PBS and fed with 90 μL of fresh medium and 10 μL of NPs solution. MTT dye (5 mg mL^−1^ dissolved in PBS) was added after 24 or 48h. Each sample was solubilized in DMSO, and the end-point absorbance of the viable cells was recorded using a spectrophotometer (BioTex Synergy HTX) at 540 nm.

The IC_50_ was calculated as the c coefficient in Hill’s function (equation (1)), approximating the MTT proliferation percentage versus concentration data using Sigma Plot 14.5.


(1) $$y=\frac{ax^{b}}{c^{b}+x^{b}}=\frac{a}{{\left(\frac{c}{x}\right)}^{b}+1}$$

### 2.4. Lactate dehydrogenase (LDH) release assay

The LDH enzyme released by dead cells can be quantified to assess the percentage of cellular death. Percentage death of A549 and BEAS-2B cells was analyzed after 24 or 48h exposure to NP solutions of increasing concentration. We used the protocol recommended by the assay manufacturer. Briefly, the media of different treatments and control groups were transferred to 96-well plates. Cells treated with 0.1% Triton-X 100 were the *Maximum* LDH positive control, while untreated cells with 10 μL of MilliQ water were the *Spontaneous* LDH control. Final absorbance values were calculated by measuring the absorbance at 490 nm subtracted by the measured absorbance at 680 nm to eliminate background abasorbance. This was done by using the Spectrophotometer Synergy HTX BioTex. The percentage LDH release cytotoxicity was calculated using Eq. (2).


(2) $$Percentage\;LDH\;release\;Cytotoxicity=\;=\frac{NP\;treated\;LDH\;Activity\;-\;Spontaneous\;LDH\;Activity}{Maximum\;LDH\;Activity\;-\;Spontaneous\;LDH\;Activity}\times 100\%$$

### 2.5. Annexin-V/propidium iodide (PI)

Details of the experimental procedure used for the flow cytometric analysis is provided in the SI section S1.5. The Annexin-V/PI kit allows the fluorescent detection of apoptotic and necrotic cells through the specific binding of FITC-labeled Annexin V to phosphatidylserine (PS), a negatively charged phospholipid located in the cytosolic side of a plasma lipid bilayer. PS re-localization from the inner to the outer bilayer membrane is the early event during apoptosis. Annexin V-FITC labeled cells were assumed early apoptotic cells, PI and Annexin V-FITC double-stained cells were late apoptosis/necrotic, while the single cell PI-positive events were nude nuclei from the necrotic cells.

We used the Annexin-V/PI kits according to the manufacturer’s instructions. Briefly, 17.5 μL of annexin dye and 35 μL of PI were suspended in 3.5 mL of Annexin buffer to prepare the stock solution. The Annexin buffer, supplied as 20 × solution, was diluted daily with water to 1 × concentration form. We prepared the Annexin V/PI solution by diluting 10 μL of stock solution in 100 μL of the Annexin buffer. The trypsinized cells were centrifuged, resuspended in 100 μL of the Annexin V/PI solution, and incubated at room temperature for 15 min in the dark. Treated (experimental) and untreated (control) cell groups were analyzed using CytoFLEX-S Flow Cytometer and CytExpert software. Annexin V FITC was detected using the exc.488nm/em.525 nm filter, and PI – with the exc.561 nm/em.610 nm filter.

### 2.6. Tetramethylrhodamine methyl ester (TMRM) assay

TMRM is a cell-permeant, cationic dye actively sequestered by living mitochondrial cells. Changes in the TMRM signal indicates disruption of mitochondrial function. The control and treated cells were incubated with 100 μL of 20 nM TMRM in PBS for 15 min at 37 °C in the dark. The working TMRM concentration was 10 nM. Before acquisition, DAPI was added to reach a concentration of 1 μg mL^−1^. The exc.561 nm/em.610 nm filter was used to detect TMRM, and exc.405/em.450 nm filter for DAPI detection. Only the DAPI −ve (live) cells were analyzed.

### 2.7. Confocal microscopy and oxidative stress studies

NP-treated cells were qualitatively analyzed for oxidative stress using Nikon-A1 scanning laser confocal microscopy (Nikon, Olympus). Treated cells were incubated in μ-slide ibidi-polymer, tissue culture treated 8-well coverslip: BEAS-2B cells in 400 μL well^−1^ of fresh BEGM medium, while A549 cells in 400 μL well^−1^ of live cell imaging solution. After the 8-h treatment, we removed the conditioned medium and washed the cells with PBS. Cells were then loaded with 10 μL of the probe cocktail (diluted in PBS) for 15 min before the live-cell imaging. Cocktail concentrations were 10 μM carboxy-H_2_DCFDA, 5 μM MitoSox, and 10 μM Hoechst 33342. Hoechst 33342 stained the nuclei of live cells. During cellular imaging, plates were kept on the stage, maintained at 37 °C using Linkam DC-60 thermo-controller, and images were captured in Galvano mode with an oil immersion (× 100 objective) lens. They were analyzed using NIS-Elements AR 4.13.04 software. Final images were merged and analyzed using free NIH Image-J software, https://imagej.net/Fiji.

### 2.8. Statistical analysis

We used GraphPad Prism (Version 9.2.0, GraphPad Software, USA, www.graphpad.com). The data were analyzed for normality with Shapiro-Wilk test. Uncertainties were standard deviations from three independent repetitions. Differences between experimental and control mean values were analyzed using two-way ANOVA followed by Tukey’s posthoc test for LDH assays, two-way ANOVA followed by Sidak’s multiple comparison tests for the Annexin-V/PI experiments, and one-way ANOVA followed by Dunnet’s multiple comparison tests for TMRM analysis. The probabilities of incorrectly concluding that differences occurred were p ≤ 0.05 up to ****p ≤ 0.0001. For further details see, section S1.6 in SI.

## 3. Results

### 3.1. Mono-NPs in ambient PM_2.5_ and smog chamber-generated SOA particles

Mono-NPs formed in smog chamber-generated SOA particles produced by the photooxidations of toluene and benzene in the presence of NO_x_. Table S1 summarizes these experiments, including the initial concentrations of reactants (aromatics and NO_x_), the particle mass concentration of SOA formed, SOA particle mass collected on filters, and mono-NPs detected in the gas and particle phases. Toluene photooxidation gave both 2NP and 4NP in the gas and particle phases, but benzene photooxidation only yielded particulate 4NP. Similar photooxidation experiments with *o*-xylene, *m*-xylene, 1,3,5-trimethylbenzene, 1,2,4-trimethylbenzene, ethylbenzene, naphthalene, 1-methylnaphthalene, 2-methylnaphthalene, and benzyl alcohol (not shown here) also produced NPs but not 2NP and 4NP. 4NP was also produced during the photooxidation of m-cresol in the presence of NO_x_. 3-NP did not occur in any other SOA particle samples.

**Fig. 1.**
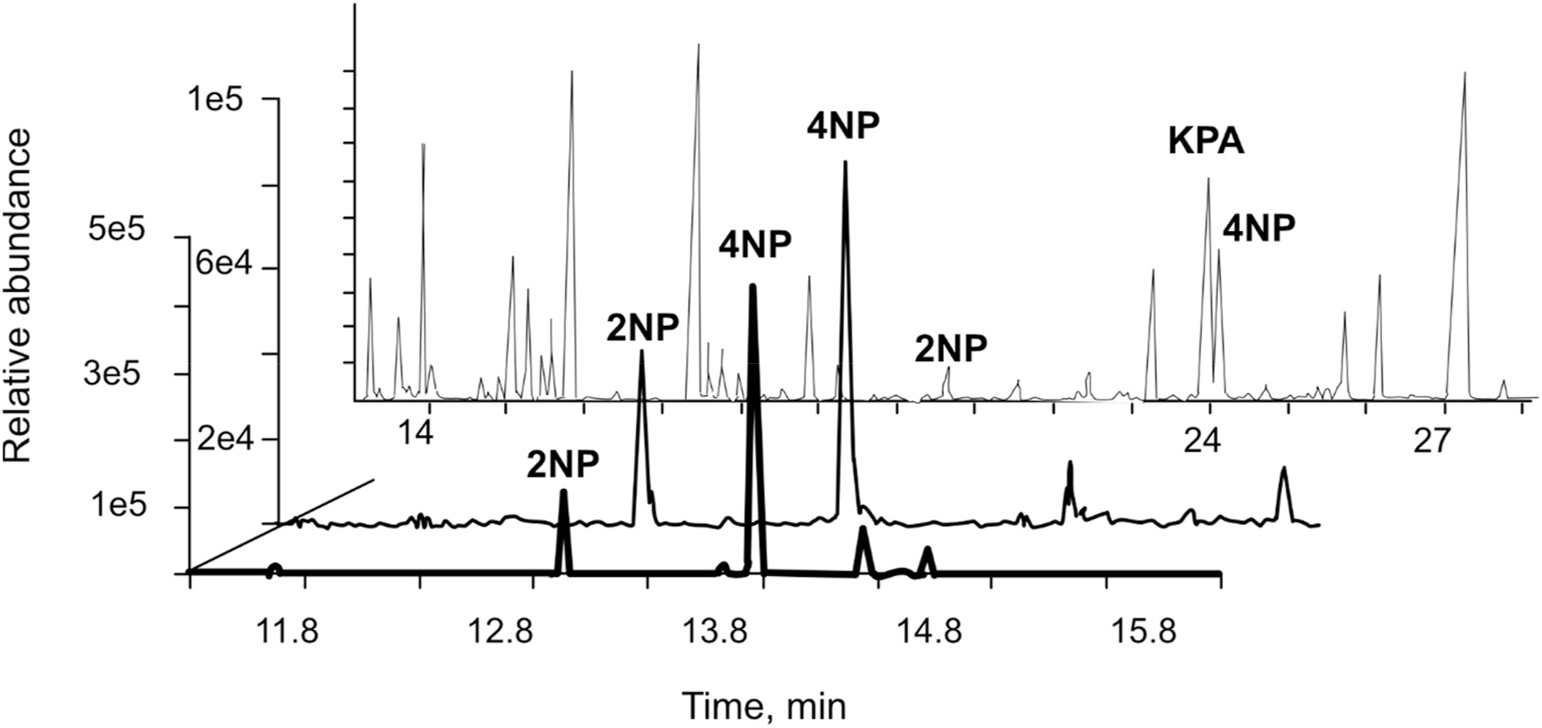
GC-MS TIC and EICs at *m/z* 212 chromatograms of the silylated samples from the photooxidation of toluene in the presence of NO_x_: (back panel) TIC of gas-phase organic sample from experiment MR092; (middle and front panels) EICs associated with ER137 experiment (gas phase and SOA particles, respectively). Retention times differ due to different chromatographic conditions.

Fig. 1 shows the total ion and extracted ion chromatograms (TIC and EICs, respectively) in the methane-chemical ionization mode (CI) associated with the gas phase and SOA particles generated from toluene photooxidation in the presence of NO_x_. EICs (middle and front panels) represent the ions with mass-to-charge ratio (*m/z*) 212 illustrating the presence of mono-NPs in the gas phase and within SOA particles, respectively. Figure S1 shows the mass spectra of trimethylsilylated 4NP obtained in EI mode (70 eV) and CI mode for the authentic standard and toluene SOA. 2NP, 3NP, and 4NP have similar mass spectra. 4NP coelutes with ketopinic acid (KPA) added as the internal standard, so EICs at *m/z* 212, 196, and 182 (Figure S1) should be used to identify 4NP.

A similar qualitative analysis was conducted for ambient PM_2.5_ collected between 2003 and 2010 at U.S. sites (Table S2). The GC-MS chromatograms recorded at the time of sample collection were now re-analyzed for the targeted NPs. 4NP was the only mono-NP observed at some sites (Table S2), mainly in winters and in some urban areas in summers. The latter is consistent with 4NP originating from atmospheric photooxidation of certain aromatics, as demonstrated by the smog chamber experiments conducted during this study.

### 3.2. Inhibitory Concentration-50 (IC_50_)

**Fig. 2.**
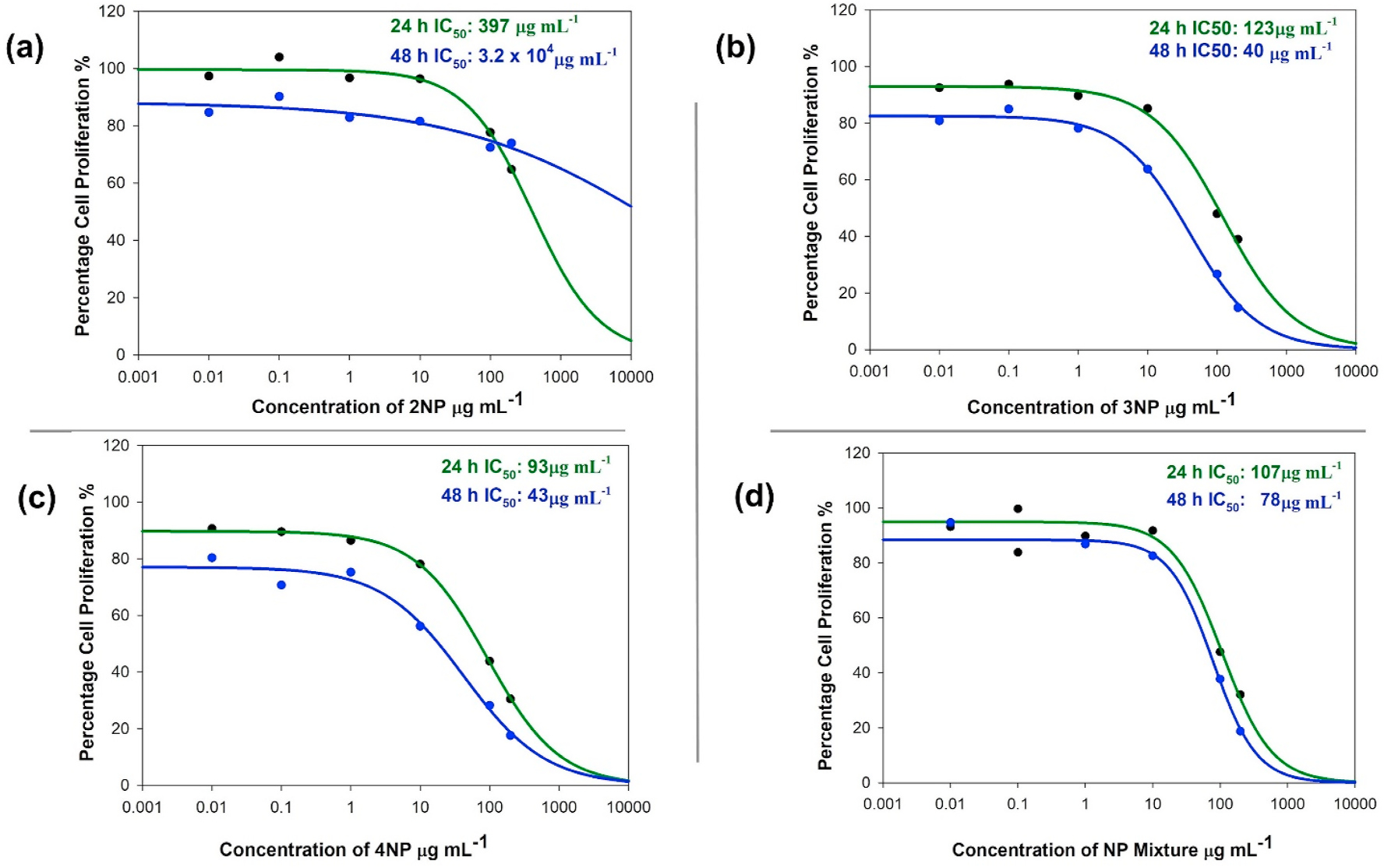
The dose-response curve of BEAS-2B cells treated with increasing concentration of NPs, as calculated through the MTT assay. The graphs show the inhibition curve generated at 24 h and 48 h exposures to: (a) 2NP; (b) 3NP; (c) 4NP; and (d) the NP mixture. Individual IC_50_ values are also provided with each graph. Similar dose response curves for A549 cells are provided in Figure S2 in SI.

Fig. 2 shows the dose-response curves for the BEAS-2B treated with 2NP, 3NP, 4NP and the NP mixture for 24 and 48 h. Similar curves for A549 cells is shown in Fig. S2 (SI). Cells were treated with increasing concentrations (i.e., 0.01–200 μg mL^−1^). Changes in cellular proliferation percentage were calculated as a function of untreated control cells at 24 and 48 h exposure. IC_50_ values determined from the curves using Hill’s approximation for treatment with 2NP, 3NP, 4NP, and their mixture were 255, 118, 89, 107 μg mL^−1^, respectively, for 24h treatments of BEAS-2B cells. Similarly, these IC_50_ values were determined to be > 10^4^, 39, 40, and 77 μg mL^−1^, respectively, for 48 h treatments of BEAS-2B cells. As shown in Fig. 2 a,b, c &d the IC_50_ values were determined to be > 10^4^, 2503, >10^4^, 223 μg mL^−1^, respectively for 24 h treatments of A549 cells, and >10^4^, 552, 552, and 122 μg mL^−1^, respectively, for 48h treatments of A549 cells. IC_50_ values were lower in BEAS-2B cells than in A549 cells for the same treatments. The inhibition curve was also different from that observed in BEAS-2B cells. For 3NP, 4NP, and the NP mixture, IC_50_ was lower for the 48 h treatments than the 24 h treatments. 2NP-treated cells had the highest IC_50_, while 4NP-treated BEAS-2B cells had the lowest IC_50_. 2NP-treated A549 cells showed no decrease in proliferation rate.

### 3.3. Cytotoxicity in NP-Treated cells

**Fig. 3.**
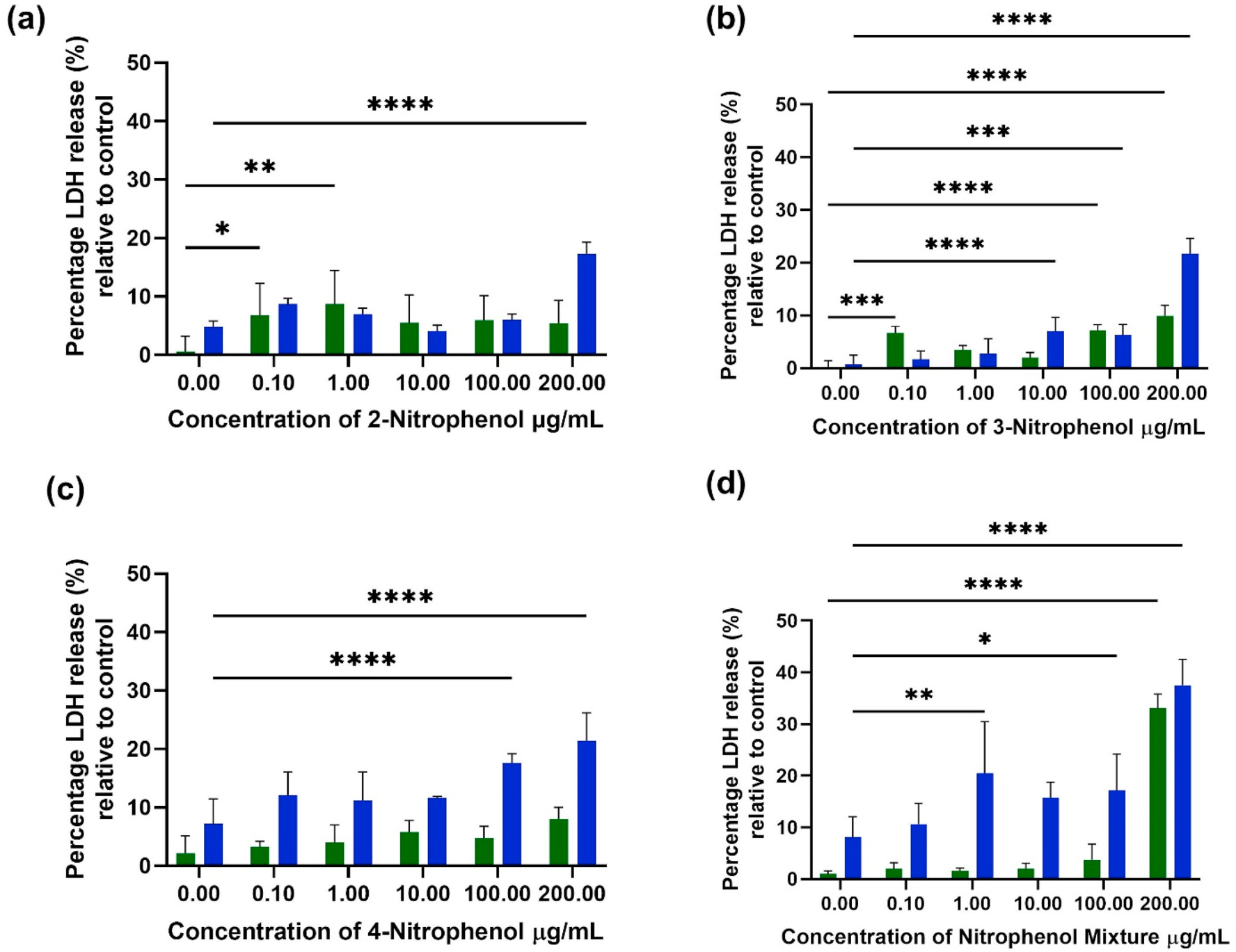
The difference in the percentage of LDH release when compared with untreated (control) BEAS-2B cells following 24 and 48 h exposure to: (a) 2NP; (b) 3NP; (c) 4NP; and (d) the NPs mixture. Results were statistically analyzed through a two-way Anova, followed by Sidak’s multiple comparison test. ANOVA probabilities of incorrectly concluding that differences occurred were: *p < 0.1, **p < 0.01, ***p < 0.001, ****p < 0.0001. Similar graphs for A549 cells can be found in Figure S3 in the SI.

Cytotoxicity of BEAS-2B and A549 cells was assessed by the LDH assay, where LDH is an enzymatic marker of cellular death (Legrand et al., 1992). Fig. 3 shows the LDH release percentage bar-graph of the BEAS-2B cells treated with 0.1–200 μg mL^−1^ of NPs, while Figure S3 shows similar data for NP-treated A549 cells relative to the untreated control cells. The results depict how increasing exposure time enhances the percentage of cellular death in each treatment type. The 2NP, 3NP and 4NP treatments (Fig. 2 a, b,c &d) exhibited 10–30% increases in LDH release when compared with untreated controls using BEAS-2B cells. The NPs mixture treatment resulted in ~30–40% increase in LDH release in BEAS-2B cells, implying that the decrease in the percentage of cellular proliferation is attributed to cellular death and not inhibition (Pucci et al., 2000).

3NP-treated A549 cells resulted in ~30–40% increase in LDH release, as shown in Figure S3. The treatment of these cells with 2NP resulted in an 18 and 10% increase in LDH release at 24 h and 48 h exposure, respectively (Figure S3a). 4NP and the NPs mixture treatment at 200 μg mL^−1^ resulted in a 10% increase in LDH release at 24 h, which increased to ~25% at 48 h exposure.

The increase in cellular death following exposure to the NPs was confirmed using live/dead stain imaging with calcein-AM that stains live cells only and propidium iodide (PI) that stains the nuclei of the dead cells. Figures S4 and S5 show the fluorescent microscopy micrographs of BEAS-2B and A549 cells treated with 200 μg mL^−1^ NPs. BEAS-2B cells treated with 2NP exhibited only a slight increased staining in the PI stain at 48 h exposure, when compared with the untreated control, with few changes in calcein-AM staining. As observed with the LDH assay, the number of dead cells increased at 48 h exposure of the BEAS-2B cells to 3NP. The cells treated with 4NP exhibited cellular growth inhibition followed by cellular death at 24 and 48 h of exposure, as can be seen by the lower number of cells found in the calcein-AM channel when compared with the untreated control at both exposure times. The slight increase in PI-stained cellular nuclei suggests cellular arrest (Wu et al., 2017), followed by the cytotoxic response after treatment of the BEAS-2B cells with 4NP. The NPs mixture caused increased cellular death after 24h exposure, showed by the increased number of PI + cells; cellular inhibition followed, which allowed for smaller numbers of live cells and high numbers of dead cells at 48 h exposure.

Changes in A549 cellular morphology were less evident than in BEAS-2B cells (Fig. S5). 2NP-treated lung cells exhibited a slight increase in PI + cells after 24 h exposure, while the cells recovered after 48 h exposure. The calcein-AM stained cells after 48 h exposures were the same as in the untreated control. 3NP-treated A549 cells exhibited cellular growth inhibition and a slight increase in PI + cells at 24 and 48 h exposure. The number of calcein-AM + cells decreased compared to the untreated control at 48 h exposure, probably due to cellular death and cellular growth inhibition (Pucci et al., 2000). A549 cells exposed to 4NP exhibited a high rate of cellular death at 48 h exposure, as demonstrated through an increased number of PI + cells in the PI channel. The A549 cells treated with NPs mixture exhibited increased PI + cells after 24 and 48 h exposure. The smaller numbers of cells in the merged channel indicates cellular death even after 24 h exposure. The calcein-AM + cells were almost negligible after 48 h exposure.

### 3.4. The mechanism of cellular death: apoptosis versus necrosis

**Fig. 4.**
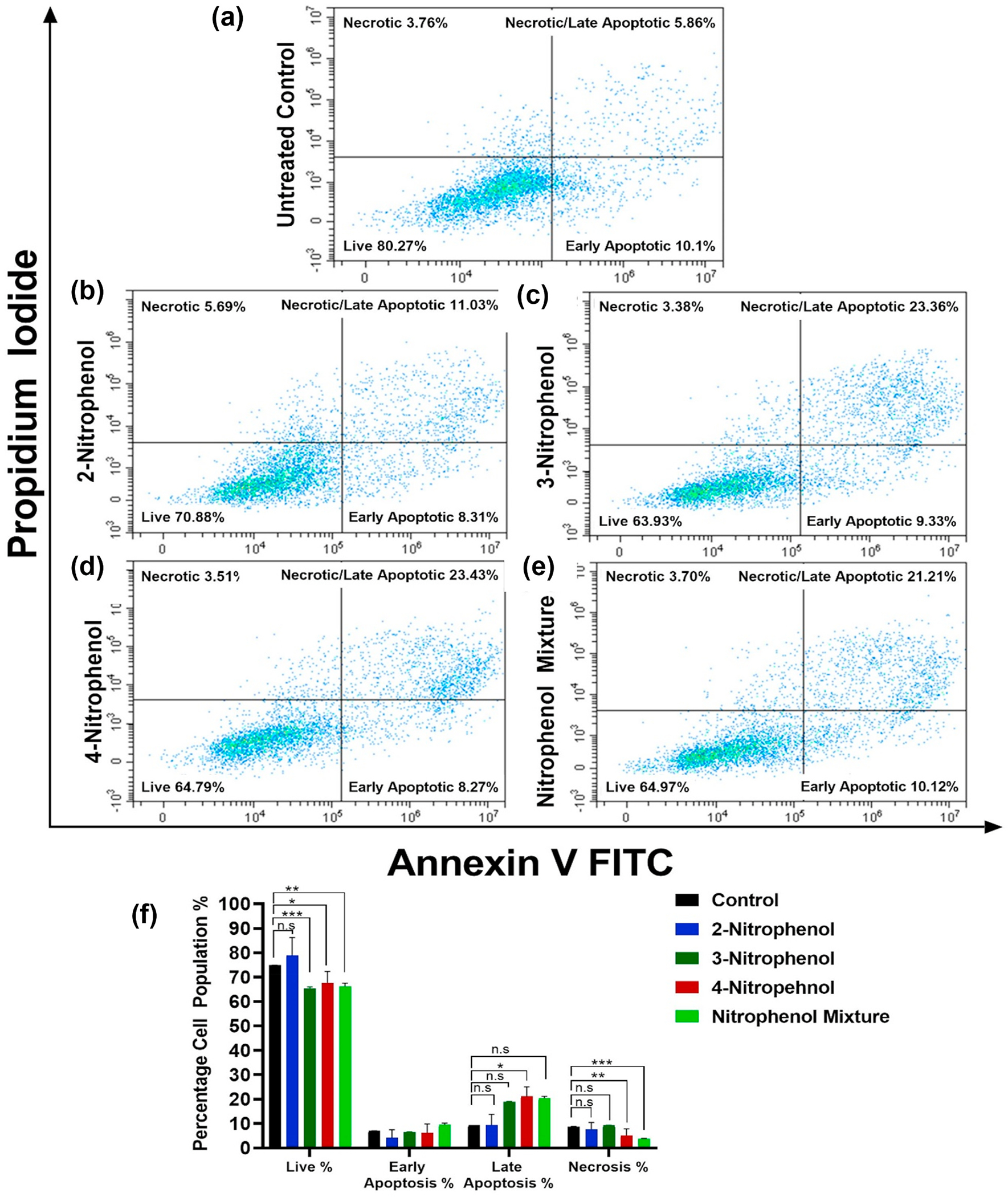
The percentages of BEAS-2B cell populations that died by various mechanisms (early apoptosis, late apoptosis/necrosis, and necrosis) or remained live after 200 μg mL^−1^ NPs exposure for: (a) untreated control; (b) 2NP; (c) 3NP; (d) 4NP; and (e) the NP equimolar mixture. Consolidated bar-graphs, determined using the Annexin-V/Propidium Iodide labeling and flow cytometry, are shown in (f). All plots shown are for the 24 h only exposures, while the plots for the 48 h exposure and the A549 cells are in SI (Figures S6S8). One-way ANOVA with Dunnett’s multiple comparisons test was used to determine the statistical significance between treatment and controls groups. ANOVA probabilities of incorrectly concluding that differences occurred were: *p < 0.1, **p < 0.01, ***p < 0.001, ****p < 0.0001.

Flow cytometry analysis with Annexin-V/FITC- and PI-labeled cells was used to elucidate the death mechanism of cells exposed to NPs. Fig. 4 shows the representative dot plots of BEAS-2B cell populations treated with 200 μg mL^−1^ of the three NPs and their equimolar mixture for 24 h; collective bar graphs show the percentage of live cells and cells that died by early and late apoptosis/necrosis and necrosis. Dot plots for other treatments of BEAS-2B cells at 48 h and all A549 cells at 24 and 48 h of exposure are shown in the SI (Figures S6, S7 and S8, respectively). All control samples were prepared with deionized water. Exposure with 3NP, 4NP, and the NP mixture decreased the number of live cells to 64–66% vs. 80% of the control cells in the 24 h experiments and 43% vs. 67% in the 48 h experiments. These same exposures increased the number of cells that died by late apoptosis/necrosis to 21–22% vs. control 6% in the 24 h experiments and 42–44% vs. 16% in the 48 h experiments. 2NP treatments did not decrease the live cell populations and marginally increased the number of cells that died by late apoptosis/necrosis.

Changes in the A549 cells were less dramatic. The 3NP, 4NP, and mixture treatments decreased the number of live cells to 69–75% vs. control 86% in the 24 h experiments, and 55–67% vs. 80% in the 48 h experiments. The number of cells that died by late apoptosis/necrosis in those treatments increased to 13–18% vs. control 6% in the 24 h experiments, and 17–28% vs. 9% in the 48 h experiments. The influence of 2NP exposures was marginal.

### 3.5. The buildup of oxidative stress following NP exposure

Oxidative stress (OS) results from the imbalance between intracellular pro-oxidant and antioxidant systems, ultimately pushing the cell towards pro-oxidant mechanisms (Martindale and Holbrook, 2002). Changes in cellular OS and the buildup of ROS (Reactive Oxygen Species) were determined using ROS-specific dyes. General ROS buildup was quantitatively analyzed using the carboxy-H_2_DCHF dye, with changes monitored every 4 h for 24 h. Figure S9 shows time-resolved results for various NP treatments of BEAS-2B and A549 cells. Carboxy-DCF fluorescence was the measure of ROS buildup in this study.

After exposure to 100 and 200 μg mL^−1^ 2NP, the general ROS signal increased significantly between 12 and 20 h of treatment in both cell lines. After 20 h, the signal decreased, implying that the cells resolved the exposure effects so that cellular death was marginal. In 3NP exposures, the ROS signal peaked after 12–16h for A549 cells and 12–20h for BEAS-2B cells. In 4NP-exposed lung cells, ROS signals peaked around 16–20 h and 12–24h in BEA-2B and A549 cells, respectively. The highest ROS occurred after exposure to the NP mixture. In BEAS-2B cells, the signal peaked 4–8h after exposure and decreased significantly after 12h. In A549 cells, the signal peaked 12–16h after exposure, and decreased significantly after 20–24h. The signals were higher in A549 cells than in BEAS-2B cells.

Figure S10 shows that MitoSox (mitochondrial superoxide) signal increased to 6–8 arbitrary units (A.U.) in BEAS-2B cells 8 h after treatment with 200 μg mL^−1^ of 3NP, 4NP, and the NP mixture. In A549 cells, the signal increased to 2 A.U. only after the mixture treatments.

**Fig. 5.**
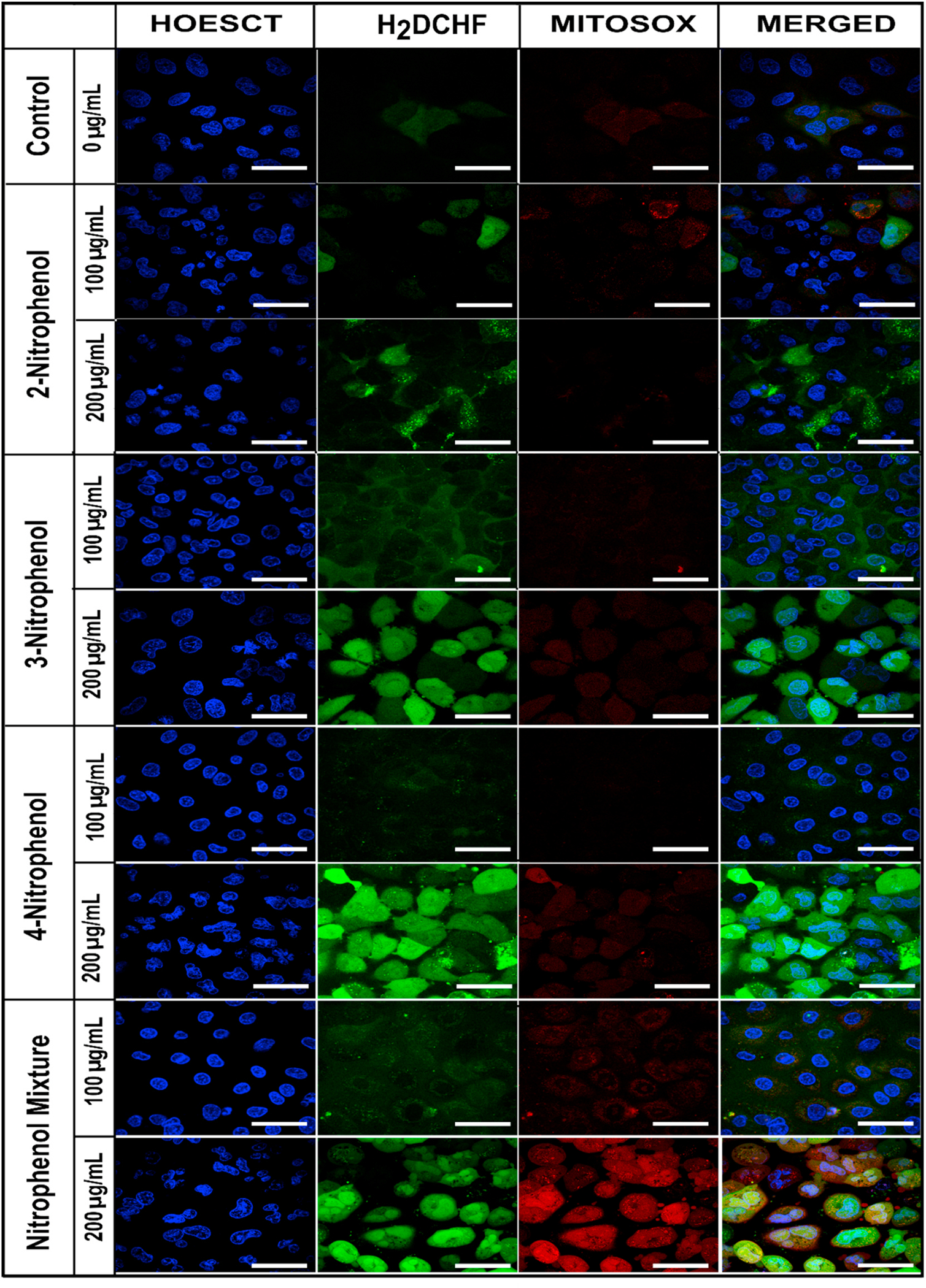
Confocal microscopy micrographs showing the OS signals following the NP treatments (100 and 200 μg mL^−1^) in) BEAS-2B cells. Blue color shows the nuclei of the live cells, green – the general ROS buildup, and red – the mitochondrial superoxide. The merged channel overlays all colors. Scale bars show 25 μm size. Micrographs for all NP treatments in A549 cells are in SI (Figure S11). (For interpretation of the references to color in this figure legend, the reader is referred to the Web version of this article.)

Fig. 5 shows the confocal microscopy micrographs of BEAS-2B cells, and Figure S11 shows similar results for A549 cells, treated 8h with NPs and their mixture. Cells were stained with ROS dyes to visualize the localization and buildup of ROS. The Hoescht dye stained the nuclei of the cells in blue, showing only the ROS signals from the live cells (also observed in the brightfield micrographs). The carboxy-DCF signals (green) highlight the regions with general ROS buildup. The MitoSox dye (red) qualitatively indicates the buildup of mitochondrial superoxide signals. The merged micrographs overlay all signals to determine if the mitochondrial superoxide signals or other ROS signals contributed to the cellular death at 24 or 48 h exposure. The increased MitoSox signals followed by the apoptotic signals indicate the possibility of intrinsic apoptotic pathway being switched-on following the exposure, and can likely be determined as the cellular toxicology mechanism (Elmore, 2007).

In the BEAS-2B cells (Figs. 5 and S10) treated with 2NP for 8h, the micrographs revealed a slight buildup of general ROS (green), while no MitoSox signals occurred. In A549 cells (Figure S11), the ROS buildup increased slightly only after the 8h NP treatments (200-μg mL^−1^).

3NP-treated BEAS-2B cells (Figs. 5 and S10) had increased general ROS and mitochondrial superoxide-associated signals, higher in 200 than 100 μg mL^−1^ treatments. In 3NP-treated A549 cells, (Figure S11), the general ROS signals increased with treatment concentration, but the MitoSox signals were absent, showing that the mitochondrial superoxide has not enhanced.

In 4NP-treated BEAS-2B cells (Figs. 5 and S10), general ROS signals increased with the treatment concentrations, while mitochondrial superoxide-associated signals occurred only in the 200-μg mL^−1^ NP treatments (Figs. 5 and S10). In A549 cells (Figure S11), general ROS signals increased only in the 200-μg mL^−1^ NP treatments.

The exposures of BEAS-2B and A549 cells to the NP mixture increased the general and mitochondrial ROS, more in 200 than 100 μg mL^−1^ treatments (Figs. 5 and S10). ROS signals and signal strengths increased when the NP mixture concentration increased from 100 to 200 μg mL^−1^. In A549 cells (Figure S11), the cells exhibited a similar increase in general- and mitochondrial-specific ROS signals, with increasing NP exposure concentrations.

### 3.6. Changes in mitochondrial membrane potential (ΔΨm)

**Fig. 6.**
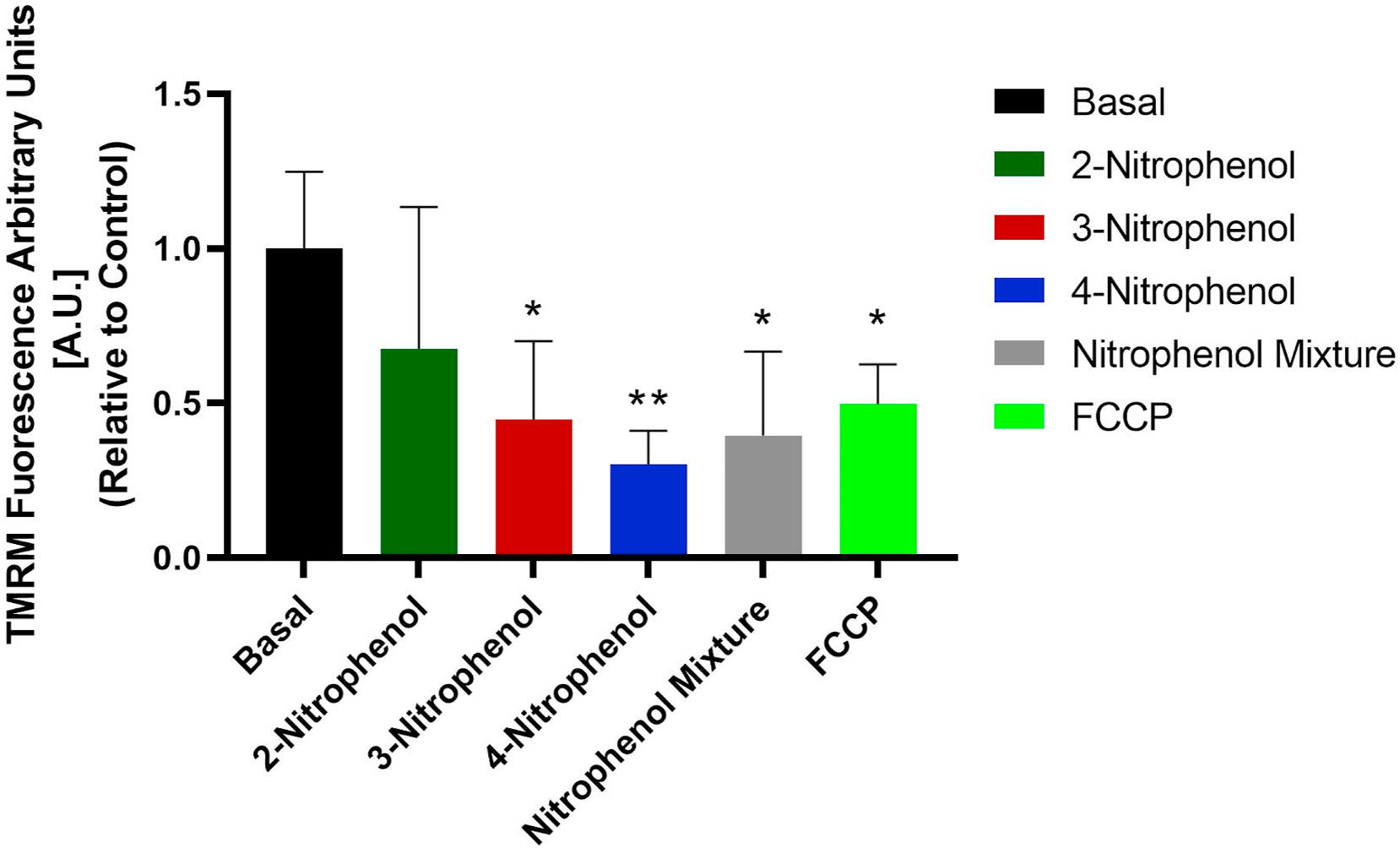
TMRM measurement of the membrane potential in BEAS-2B cells treated for 24 h with the 200-μg mL^−1^ of 2NP, 3NP, 4NP, and their equimolar mixture. Treatments with carbonyl cyanide 4-(trifluoromethoxy) phenylhydrazone (FCCP) were a positive control for ΔΨm decrease. ANOVA probabilities of incorrectly concluding that differences occurred were: *p* < 0.05 and **p ≤ 0.01.

TMRM is a cell-permeant dye sequestered by active mitochondria to measure the mitochondrial membrane potential (ΔΨm) (Chazotte, 2011). Changes in ΔΨm provide essential information on mitochondrial health. The decrease in ΔΨm indicates a collapse of the proton gradient in a mitochondrial membrane (Zorova et al., 2018). An increase in mitochondrial superoxide and decrease in ΔΨm may indicate mitochondrial dysfunction after NP exposures; the exposure inadvertently “switches on” the intrinsic apoptotic pathway (Elmore, 2007). Fig. 6 shows that 24 h exposure of BEAS-2B cells to 3NP, 4NP, and the NP mixture decreased TMRM signals compared to the untreated controls (basal). 2NP exposure did not change the ΔΨm signal. The NP-treated A549 cells (Figure S12) showed no change in the ΔΨm signals. The positive control treatments with FCCP decreased ΔΨm signals in both types of cells.

## 4. Discussion

This study shows that mono-NPs occur in the field and lab-generated aerosol particles and evaluates their toxicological profiles in human lung cells. The NPs concentrations in atmospheric samples varied significantly (Tables S3S6). 4NP, which forms by the photochemical oxidations of toluene and benzene in the presence of NO_x_, is the most abundant NP we characterized in PM_2.5_. In ambient air, 2NP concentrations were previously measured from 0.008 to 1.139 μg m^−3^, and 4NP concentrations were previously measured from 0.040 to 1.400 μg m^−3^ (Belloli et al., 1999; Belloli et al., 2006; Rubio et al., 2012). The concentration of particle-bound 2NP in the air was 0.0001–10.86 μg m^−3^ (Lanzafame et al., 2021), and the concentration of particle-bound 4NP was 0.0002–0.768 μg m^−3^ (Kitanovski et al., 2020b; Li et al., 2016). Rainwater samples contained 0.026–1.4 μg dm^−3^ of 2NP (Harrison et al., 2005; Leuenberger et al., 1985; Rippen et al., 1987), and 0.1–19.5 μg dm^−3^ of 4NP (Harrison et al., 2005; Leuenberger et al., 1985; Rippen et al., 1987). In cloud water, 2NP concentrations were 0.024–0.200 μg dm^−3^ (Harrison et al., 2005; Lüttke et al., 1999), and 4NP concentrations were 1.66–16.27 μg dm^−3^ (Harrison et al., 2005; Levsen et al., 1993). Exceptionally high concentrations of 2NP and 4NP s in dew occurred in Santiago de Chile: up to 237 μg dm^−3^ and 629 μg dm^−3^, respectively (Rubio et al., 2012). Fog water contained only 0.0004–0.0012 μg dm^−3^ of 4NP (Harrison et al., 2005; Richartz et al., 1990). 3NP was rarely detected: 0–147 μg dm^−3^ in dew in Santiago de Chile (Rubio et al., 2012), and 0–0.003 μg m^−3^ total in air and PM at various European sites (Harrison et al., 2005; Wennrich et al., 1995).

We evaluated toxicological profiles of 2NP, 3NP, 4NP, and their equimolar mixture in terms of cytotoxicity, OS, changes in the ΔΨm, and apoptosis. Previously, we showed that lung cell membranes exposed to the equimolar NP mixture (100–200 μg mL^−1^) underwent disruption (Majewska et al., 2021). The IC_50_ values of the NPs determined here for two cell lines help estimate the inhalation safety indices. In our previous study we calculated that the 200 μg mL^−1^ concentration corresponds to 62.5 μg cm^−2^ of cellular exposure, and thus, a similar approach was adopted herein to estimate NP exposures to cells, assuming negligible lung clearance rates (summarized in Table S7) (Khan et al., 2021). Furthermore, the multiple path particle dosimetry (MPPD) model by Gangwal et al. (2011) was used to estimate the lung uptake of ultrafine particles (≤100 nm diameter). We predicted the uptake ranges from 0.006 to 0.02 μg cm^−2^ to a particle concentration of 100 mg m^−3^ of these particle sizes at a 24 h exposure time. The estimated lung uptake was assumed to be 7.5 × 10 ^−4^ μg cm ^−2^ in the particles “hot spot” regions (Khan et al., 2021). IC_50_ values were lower in BEAS-2B cells than in A549 cells, implying BEAS-2B cells were more sensitive to NP exposures, consistent with the literature (Khan et al., 2021; Zhang et al., 2017). Continuous exposure to 3NP and 4NP at the IC_50_ concentration found for BEAS-2B cells may cause lung inhibition in ~47 and 51 years, respectively (Paur et al., 2011). A similar effect of 2NP and the NP mixture requires exposure exceeding the human lifetime; hence, we assume those pollutants are relatively safe. However, NP-containing PM_2.5_ may not be safe due to syngery with other chemical components in PM_2.5_ and warrants further study. For instance, SOA from α-pinene ozonolysis was more toxic than its components (Khan et al., 2021).

The NPs examined here exhibited various inhibitory and cytotoxic effects in both cell lines. Individually, 4NP was the most cytotoxic, and 2NP was the least toxic. Similar differences occurred for two isomeric nitro-polycyclic aromatic hydrocarbons (Øya et al., 2011). The NP mixture showed an antagonist effect of its components (Lin et al., 2004), as its IC_50_ value was higher than that of 3NP and 4NP yet lower than the 2NP value in both cell types. The cytotoxic assessment revealed mixed inhibition and cytotoxic effects in the exposed cells. For 4NP exposures, the cellular inhibition occurred in the first 24 h of exposure, followed by increased cellular death between 24 and 48 h, suggesting that high ROS and organelle dysfunction induced cellular death.

Apoptosis (programmed cell death) is an energy-dependent biochemical mechanism different from necrotic death, a toxic, energy-independent process is one of the most studied cellular death mechanisms (Elmore, 2007), and is morphologically different than necrotic cellular death. The mechanism of cellular death (apoptosis) was the same in the 3NP-, 4NP- and NP mixture-treated cells; however, BEAS-2B cells treated with 3NP underwent apoptosis within 24 h of exposure, while 4NP-treated BEAS-2B and A549 cells increased in necrosis/late apoptosis between 24 and 48h. Generally, during *in vitro* exposures, necrosis follows late apoptosis (Fink and Cookson, 2005). That effect by 4NP was the strongest of all NPs. Thus, cellular exposures to 3NP, 4NP, and the NP mixture activated the internal apoptotic pathways (Elmore, 2007). The time- and dose-dependent apoptotic and intracellular ROS build-up effects are seen with previous atmospheric PM, PM_2.5,_ PM_0.3,_ and black carbon emissions (An et al., 2019; Badran et al., 2020), implying disruption in the intracellular ROS and mtROS system to induce apoptosis through an “intrinsic pathway.” We showed that exposure to the NP mixture caused changes in cell membranes (Majewska et al., 2021); specifically, the internalization of NPs took place to exhibit molecular-level cellular changes. Hence, the induction of apoptosis via activation of internal apoptotic pathway (Elmore, 2007). Such changes can occur in mitochondrial membranes, which, biochemically, have a similar structure to the cell membranes (Kühlbrandt, 2015). Changes in ΔΨm we observed confirm that conclusion.

Lung epithelial cells are vulnerable to endogenous and exogenous oxidants, including air pollutants (Wright et al., 1994). ROS induced by the imbalance in antioxidant and oxidant regulations caused by air pollutants may cause adverse lung effects following exposure to urban pollutants (Oh et al., 2011). The persistent induction of ROS and pro-survival responses may contribute to the progression of diseases following exposure to atmospheric PM_2.5_ (Merk et al., 2020). Cells exposed to 2NP, 3NP, 4NP, and their equimolar mixture exhibited increased general ROS. However, cells exposed to 200 μg mL^−1^ 2NP cells recovered within 24 h exposure. ROS, which include free radicals (e.g., OH and O_2_^•−^) and nonradicals (e.g., H_2_O_2_), can increase OS within cells and force mitochondria to promote cellular apoptosis (Badran et al., 2020). Cells exposed to 100 and 200 μg mL^−1^ of 3NP, 4NP, or NP mixture peaked with ROS after 12–20 h and underwent apoptosis after 24 h.

The buildup of mitochondrial O_2_^•−^ indicates the ROS imbalance, as mitochondria are important modulators and regulators of oxidation-reduction processes (Piantadosi and Suliman, 2017). Besides, moderately reactive O_2_^•−^ is detoxified by mitochondrial superoxide dismutases to less toxic H_2_O_2_ (Oh et al., 2011). The mtROS buildup indicates that mitochondrial dysfunction may increase general ROS signals, impair electron transport, and decrease the mitochondrial membrane potential (Liu and Chen, 2017). BEAS-2B cells treated with 3NP, 4NP, and the NP mixture showed significant mtROS, implying collapse of mitochondrial membrane potential that ultimately induced intrinsic apoptosis. The ROS buildup in A549 cells was lower than in BEAS-2B cells, probably due to different metabolic, proliferative, and higher “respiratory” effects in cancer cell lines (Homma et al., 2009; Wang et al., 2019). Dysmorphic mitochondria are associated with chronic respiratory diseases, including asthma, allergy, bronchitis, and pulmonary hypertension (Rowlands, 2016). Thus, the NP exposure effects at sub-organelle levels may indicate disruptions in mitochondrial membrane functioning, warranting future chronic exposure studies using IC_50_ concentrations to resolve underlying lung pathophysiology.

## 5. Conclusions

Mono-NPs are pollutants that are present in ambient PM_2.5_ and smog chamber-generated SOA particles. The cellular growth inhibition following exposures to NPs correlates with intracellular ROS and mtROS buildup. Significantly, BEAS-2B cells treated with 3NP and 4NP showed the highest growth inhibition and mtROS buildup, followed by apoptosis between 24 and 48 h of exposure. 4NP was the most abundant NP in environmental and smog-chamber samples and caused the highest toxicological response. IC_50_ values show that NPs are most dangerous in high-concentration scenarios such as wildfires, industrial facilities, or smog events. Our results suggest that acute atmospheric exposures to NPs may be toxic at high concentrations, but not at typical ambient PM_2.5_ concentrations (i.e., 0.0012–16.26 μg dm^−3^). Future studies of chronic exposure to particulate NPs are warranted, especially *in vivo* as real lungs are more complex than the *in vitro* system used in this study.

## Supplementary Material

Supplement1
