# Supplementary material for: Cytotoxicity and oxidative stress induced by atmospheric mono-nitrophenols in human lung cells
Source: Environ Pollut. Author manuscript; Available in PMC 2023 May 15. (PMC9171836; doi:10.1016/j.envpol.2022.119010)
Supplement: Supplement1 [file NIHMS1804330-supplement-Supplement1.docx]

**Supporting Information**

**Cytotoxicity and Oxidative Stress Induced by Atmospheric Mono-Nitrophenols in Human Lung Cells**

Faria Khan,^1,^ Mohammed Jaoui,^2^ Krzysztof Rudziński,^1^ Karina Kwapiszewska,^1^ Alicia Martinez-Romero,^3^ Domingo Gil-Casanova,^3^ Michael Lewandowski,^2^ Tadeusz E. Kleindienst,^2^ John H. Offenberg,^2^ Jonathan D. Krug,^2^ Jason D. Surratt^4,5^ and Rafal Szmigielski^1,^*

^1^ Institute of Physical Chemistry, Polish Academy of Sciences, Kasprzaka 44/52, 01-224 Warsaw, Poland

^2^ Center for Environmental Measurement & Modeling, U.S. Environmental Protection Agency, Research Triangle Park, NC 27711, United States of America.

^3^ Cytomics Core Facility. Príncipe Felipe Research Center. Avda. Eduardo Primo Yúfera, 3, 46012, Valenica, Spain

^4^ Department of Environmental Sciences and Engineering, Gillings School of Global Public Health, University of North Carolina at Chapel Hill, Chapel Hill, North Carolina, United States 27599

^5^ Department of Chemistry, University of North Carolina at Chapel Hill, Chapel Hill, North Carolina, United States 27599

* [ralf@ichf.edu.pl](mailto:ralf@ichf.edu.pl)

**Section S1 Supplementary Methodology**

**S1.1 Cell culture and medium**

BEAS-2B (ATCC^®^ CRL-9609™) and A549 (ATCC^®^CCL-185™) cell lines were purchased from ATCC.  For details of culture and medium conditions see (Khan et al., 2021). Briefly, for BEAS-2B cells, we used bronchial epithelial cell growth medium (BEGM), with supplements and growth factors (BEpiCM, ScienCell, USA), recommended by ATCC. A549 cells were cultured in Dulbecco's modified eagle's medium (DMEM, Institute of Immunology and Experimental Technology, Wrocław, Poland) or in phenol-red free DMEM in heat-inactivated fetal bovine serum (FBS, 5% or 10%), L-glutamine (L/G, 2 mM) and penicillin-streptomycin (P/S, 100 mg ml^-1^). Trypsin-EDTA (0.25% solution with phenol red, Sigma-Aldrich, USA) was used to detach the adherent cells for passaging and maintenance at 37 ºC in a 5% CO_2_ humidified incubator. The BEAS-2B cell lines were maintained between 3-20 passages, while A549 cells were maintained between 3-30 passages during experiments (Schulz et al., 2002).

**S1.2- Calcein-AM/ Propidium Iodide Stain Imaging**

Live-cell imaging using calcein-AM (1mg mL^-1^ dissolved in DMSO for live cells) and propidium iodide (1 mg mL^-1^ in DI water for dead cells) was performed according to the protocol and microscope settings provided elsewhere (Khan et al., 2021). BEAS-2B and A549 cells treated with nitrophenols (NPs) were stained with the calcein-AM: PI in ratio (1:10) where final concentration in the sample was 100 µg mL^-1^ for PI, and 1 µg mL^-1^ for calcein-AM, dissolved in PBS, and imaged at 24 and 48 h of treatments. The concentration of NPs used was 200 µg mL^-1^ in the experiment. Images were acquired using fluorescence microscope (Nikon Eclipse T1-SAM, Japan) in TRITC and FITC filter range (Ti-FLC filters) at x400 magnification. Nikon DS-U2 digital sight (Japan) high-resolution camera was used to image the apoptotic bodies and dead cell DNA in the treated cells. NIS-Elements imaging software was used for initial imaging, while final images were analyzed with freely available software, including the GIMP image editor <https://www.gimp.org/> and NCBI’s Image-J software at <https://imagej.net/Fiji>.

**S1.3- General Oxidative Stress determination using Spectrophotometer.**

Carboxy-H_2_DCFDA is a cell permeant probe, which, upon deacetylation by cellular sterases and oxidation by ROS, is converted into green, fluorescent carboxy-DCF. The BEAS-2B and A549 cells were seeded to the 96-well plate at least 16 h prior to the exposure. Then, conditioned medium was discarded, and cells resuspended in 100 µL of cell culture medium. 100 µL of PBS with 20 µM of carboxy-H_2_DCFDA were subsequently added and incubated for 15 min at 37 ºC in the dark. Working carboxy-H_2_DCFDA concentration was 10 µM. The exc.488 nm/em.525 nm filter was used to detect carboxy-DCF signal using fluorescent spectrophotometer at 4-hour intervals up to 24 hours of treatment.

**S1.4- Mitochondrial Specific Superoxide determination using Flow Cytometer.**

MitoSox is a cell permeant fluorogenic dye that produces red fluorescence in the presence of mitochondrial-specific superoxide. To BEAS-2B and A549 control and treated cells resuspended in 100 µL of cell culture medium, 100 µL of PBS and 10 µM of MitoSox were added and incubated for 15 minutes at 37ºC in the dark. Working MitoSox concentration was 5 µM. Before the acquisition, DAPI was added to a final concentration of 1 µg mL^-1^. The exc.561 nm/em.610 nm filter was used to detect MitoSox, and exc.405/em.450 nm filter for DAPI detection.

**S1.5** **- Statistical Analyses: One-Way and Two-Way Anova**

The fold changes relative to untreated control for MitoSox and carboxy-H_2_DCHFA were analyzed using one-way Anova followed by Dunnet’s multiple comparison tests using Graph-pad Prism 9.1. The values of p≤0.05 were considered significant in each of the tests and values of up to ****p≤0.0001 significance was reported.

**Section S2: Supplementary Results**

**Table S1.** Initial reactants conditions of hydrocarbon (HC) and nitrogen oxides (NO_x_) as well as the mass of SOA (mg) collected on the filters and analyzed by GC-MS for selected representative smog chamber experiments. PP: particle phase; GP: gas phase; 2NP: 2-nitrophenol; 4NP: 4-nitrophenol; S: experiment conducted in static mode; D: experiment conducted in dynamic mode. Ammonium sulfate seed aerosol at 1 µg m^−3^ was used. The initial NO_x_ during the irradiations was greater than 98% NO.

| Exp. ID | HC | Initial HC (ppmC) | Initial NO_X_ (ppb) | SOA mass (mg) /[SOA] (µg m^-3^) | Phase  analyzed | Detected  NPs |
| --- | --- | --- | --- | --- | --- | --- |
| ER137^S, *^ | Toluene | 5.44 | 293 | 8.85/54.95 | GP; PP | 2NP; 4NP |
| ER-343^D^ | Benzene | 19.70 | 259 | 3.69/182.43 | PP | 4NP |
| MR092 | Toluene | 18.60 | 810 | 1.77/595.56 | GP; PP | 2NP; 4NP |

**Table S2.** Ambient PM_2.5_ samples analyzed qualitatively in this study for mono-NPs. Only 4NP was detected, whereas 2NP and 3NP were not.

| Date | Study Location | Site type/ landscape | 4NP detected |
| --- | --- | --- | --- |
| Mar–Sep, 2003 | Research Triangle Park, NC | Semi-rural | No |
| Mar–Sep, 2004 | LADCO: Bondville, IL | Rural | Yes |
| Mar–Sep, 2004 | LADCO: Cincinnati, OH | Urban | No |
| Mar–Sep, 2004 | LADCO: Detroit, M | Urban | No |
| Mar–Sep, 2004 | LADCO: E. St. Louis, IL | Industrial | No |
| Mar–Sep, 2004 | LADCO: Northbrook, IL | Suburban | Yes |
| May–Aug, 2005 | SEARCH: Atlanta, GA | Urban; residential | No |
| May–Aug, 2005 | SEARCH: Birmingham, AL | Industrial; residential | No |
| May–Aug, 2005 | SEARCH: Centreville, AL | Rural | No |
| May–Aug, 2005 | SEARCH: Pensacola, FL | Suburban | No |
| Aug, 2005 | SOAR: Riverside, CA | Urban | Yes |
| Mar–Sep, 2006 | Research Triangle Park, NC | Semi-rural | No |
| Jul–Aug, 2009 (summer) | CMAPS: Cleveland, OH | Industrial | No |
| Jul–Aug, 2009 (summer) | CMAPS: Medina, OH | Rural | No |
| Feb, 2010 (winter) | CMAPS: Cleveland, OH | Industrial | Yes |
| Feb, 2010 (winter) | CMAPS: Medina, OH | Rural | Yes |
| May–Jun, 2010 | CalNex: Bakersfield, CA | Urban; industrial | Yes |
| May–Jun, 2010 | CalNex: Pasadena, CA | Urban; industrial | Yes |

**Table S3**. Concentration of 2-nitrophenol (2NP) and 4-nitrophenol (4NP) in atmospheric compartments, including gas phase (µg m^-3^) and aqueous phase (µg dm^-3^).

| **Place** | **Phase** | **2NP** | | | **4NP** | | | **Year** | **Ref.** |
| --- | --- | --- | --- | --- | --- | --- | --- | --- | --- |
|  |  | **Min** | **Max** | **Av.** | **Min** | **Max** | **Av.** |  |  |
| **Air** | | | | | | | | | |
| Milano | Gas |  |  |  |  |  | 0.3 |  | (Belloli et al., 1999; Harrison et al., 2005) |
| Milano, center | Gas | 0.130 | 0.177 | 0.139 | 0.042 | 0.163 | 0.085 | Summer 1999 | (Belloli et al., 2006) |
| Milano, tunnel | Gas | 0.232 | 1.139 | 0.641 | 0.387 | 0.993 | 0.642 | Summer 1999 | ibid |
| Lombardia, urban | Gas |  |  |  | 0.094 | 0.163 | 0.118 | Winter 1999 | ibid |
| semi-rural | Gas |  |  |  |  |  | 0.087 |  | ibid |
| rural |  |  |  |  |  |  | 0.042 |  | ibid |
| Rome, city park | Gas |  |  | 0.01 |  |  | 0.004 | Spring 2003 | (Cecinato et al., 2005) |
| Santiago, university | Gas | 0.008 | 0.106 | 0.114 | 0.040 | 1.40 | 462 | Sum 2010, 2011 | (Rubio et al., 2012) |
| Iowa city | Gas |  |  |  |  |  | 0.001 | 2015 | (Al-Naiema and Stone, 2017) |
| **Clouds** | | | | | | | | | |
| Great Dunn Fell | Gas | 0.001 | 0.008 |  | 0.0001 | 0.02 |  | May 1976 | (Harrison et al., 2005; Lüttke et al., 1999) |
|  | Aqu | 0.024 | 0.2 |  | 0.007 | 2.9 |  |  | ibid |
|  | Aqu |  |  | 0.2 |  |  | 2.2 | April 1993 | ibid |
| Mount Brocken | Gas | 0.01 | 0.50 |  |  |  |  | 1994 | ibid |
|  | Aqu |  |  | 0.03 |  |  | 21 |  | ibid |
| Vosges mountains | Aqu |  |  |  | 1.66 | 16.27 | 5.46 | 1991 | (Harrison et al., 2005; Levsen et al., 1993) |
| **Rain** | | | | | | | | | |
| Portland, OR | Gas | 0.011 | 0.039 | 0.025 |  |  |  |  | (Harrison et al., 2005; Leuenberger et al., 1985) |
|  | Aqu | 0.026 | 0.130 | 0.059 |  |  |  |  |  |
| Dübendorf, CH | Gas |  |  | 0.35 |  |  |  | 1985 | (Harrison et al., 2005; Leuenberger et al., 1988) |
|  | Aqu |  |  | 0.60 |  |  |  |  |  |
| Vosqes mountains | Aqu |  |  |  | 1.11 | 7.57 | 2.95 | 1991 | (Harrison et al., 2005; Levsen et al., 1993) |
| Hannovwr | Aqu | 0.03 | 0.68 | 0.18 | 1.2 | 19.5 | 5.7 | 1990 | (Harrison et al., 2005; Levsen et al., 1990) |
| Western Germany | Aqu | 0.1 | 1.4 |  | 2 | 16 |  |  | (Harrison et al., 2005; Rippen et al., 1987) |
| Frankfurt a M. storm | Aqu |  |  |  |  |  | 24 |  | ibid |
| Western Germany | Aqu |  |  |  | 0.1 | 1.2 |  |  | (Rippen et al., 1987) |
| Lombardia | Aqu |  |  |  | 12 | 29 |  |  | (Belloli et al., 2006) |
| Roskilde | Aqu |  |  |  | 1 | 11.9 | 3.5 |  | (Asman et al., 2005) |
| Oure | Aqu |  |  |  | 0.1 | 5.4 | 1.2 |  | ibid |
| **Fog** | | | | | | | | | |
| Ochswnkopf Mt. | Aqu |  |  |  | 0.0004 | 0.002 | 0.001 | 1988 | (Harrison et al., 2005; Richartz et al., 1990) |
| Bayreuth Uni | Aqu |  |  |  | 0.0007 | 0.0012 | 0.001 | 1988 | ibid |
| **Dew** | | | | | | | | | |
| Santiago Uni | Aqu | Nd | 237 | 25 | Nd | 629 | 71 | Summer 2011 | (Rubio et al., 2012) |
| Snow |  |  |  |  |  |  |  |  |  |
| Mount Melbourne  (Antarctic) | Aqu |  |  | < LOD | < LOD | 0.013 | 0.008 |  | (Harrison et al., 2005; Vanni et al., 2001) |

**Table S4**. Concentrations of 3-nitrophenol (3NP) in various environmental compartments, including the gas/particle phases (µg m^-3^) and aqueous phase (µg dm^-3^)

| **Place** | **Phase** | **3NP** | | | **Year** | **Ref.** |
| --- | --- | --- | --- | --- | --- | --- |
|  |  | **Min** | **Max** | **Av.** |  |  |
| Santiago Uni | Dew (aqu) | Nd | 147 | 8 | Summer 2011 | (Rubio et al., 2012) |
| Strassvourg, urban | Air + PM | Nd | 0.003 | 0.0001 |  | (Delhomme et al., 2010) |
| Schiltgheim, suburban | Air + PM | Nd | 0.002 | 0.0002 |  |  |
| Erstein, rural | Air + PM | Nd | 0.00005 | 0.000001 |  |  |
| Elsnig, ground water, lower level | Aqu |  |  | 55 |  | (Harrison et al., 2005; Wennrich et al., 1995) |

**Table S5**. Concentrations of 2NP and 4NP in ambient particulate matter (µg m^-3^)

| **Place** | **Phase** | **2NP** | | | **4NP** | | | **Year** | **Ref.** |
| --- | --- | --- | --- | --- | --- | --- | --- | --- | --- |
|  |  | **Min** | **Max** | **Av.** | **Min** | **Max** | **Av.** |  |  |
| NRI Kanagawa |  | 3.4 ^a^ | 3.9 ^a^ |  | 5.1 ^a^ | 42 ^a^ | 14.48 ^a^ |  | (Nojima et al., 1983; Richartz et al., 1990) |
| Hong Kong urban | PM_2.5_ |  |  |  | 0 | 0.009 | 0.001 |  | (Chow et al., 2016) |
| Detling, UK | PM_0.13_ |  |  |  |  |  | 0.00002 |  | (Chow et al., 2016; Mohr et al., 2013) |
| Ljubljana, urban | PM_10_ |  |  |  | 0.0005 | 0.004 | 0.002 | Winter 2010-2011 | (Kitanovski et al., 2012) |
|  |  |  |  |  | 0.0001 | 0.002 | 0.0002 | Summer 2010-2011 | ibid |
| Hamilton, Ontario | PM_1_ |  |  |  | 0.0002 | 0.002 | 0.0007 | 2000 | (Irei et al., 2017) |
| Simcoe, Ontario | PM_1_ |  |  |  | < LOD | 0.002 | 0.0004 | 2000 | ibid |
| Toronto, tunnel | PM_1_ |  |  |  | 0.003 | 0.025 | 0.009 | 2000 | ibid |
| Iowa City | PM_2.5_ |  |  |  | 0.0006 | 0.0005 |  | 2015 | (Al-Naiema and Stone, 2017) |
| Mainz | PM_10_ |  |  |  | 0.0002 | 0.001 | 0.0008 |  | (Kitanovski et al., 2020b) |
| Thesaloniki | PM_10_ |  |  |  | 0.0008 | 0.004 | 0.0026 |  | ibid |
| Shanghai |  |  |  |  | 0.151 | 0.768 | 0.304 |  | (Li et al., 2016) |
| Birmingham | PM_2.5_ | 0.0001 | 0.0016 |  | 0.0001 | 0.0004 |  | 2007-2008 | (Özel et al., 2011) |
| Melpitz |  |  |  | < LOD | 0.0008 | 0.0010 |  | 24 Jan 2012 | (Teich et al., 2014) |
|  |  |  |  |  | 0.0001 | 0.0001 |  | 30 Aug 2012 | ibid |
|  |  |  |  |  | 0.0005 | 0.0006 |  | 10 Oct 2012 | ibid |
| Mainz | PM_10_ | < LOD | 0.009 | 0.002 | < LOD | 0.010 | 0.002 | Summer 2007 | (Zhang et al., 2010) |
|  | TSP | < LOD | 0.009 | 0.002 | < LOD | 0.013 | 0.004 | “ | ibid |
| Rome, city park | PM_5_ |  |  | 0.0035 |  |  | 0.0178 | Spring 2003 | (Cecinato et al., 2005) |
| Paris |  | < 0.0001 | 10.863 | 0.0527 | < 0.0001 | 0.0279 | 0.0069 | 2014-2015 | (Lanzafame et al., 2021) |
| Diesel | PM_2.5_ |  |  | nd |  |  | 0.004 |  | (Yassine et al., 2020) |
| Marine Diesel | PM_2.5_ |  |  | nd |  |  | 0.011 |  | ibid |
| NIST 1649b urban | PM |  |  | < LOD |  |  | 0.0024 |  | ibid |
| NIST 1650b Diesel | PM |  |  | < LOD |  |  | 0.072 |  | ibid |
| Kladno, winter | PM_10_ |  |  |  |  |  | 0.00554 |  | (Kitanovski et al., 2020a) |
|  | PM_2.5_ |  |  |  |  |  | 0.00301 |  | ibid |
| Ostrawa, winter | PM_10_ |  |  |  |  |  | 0.00906 |  | ibid |
|  | PM_2.5_ |  |  |  |  |  | 0.00535 |  | ibid |
| Ostrava, summer | PM_10_ |  |  |  |  |  | 0.00112 |  | ibid |
|  | PM_2.5_ |  |  |  |  |  | 0.00085 |  | ibid |

^a^ mg g^-1^

**Table S6**. Concentration of 2NP and 4NP in aquatic systems (µg dm^-3^, aqueous phase).

| **Place** | **2NP** | | | **4NP** | | | **Ref.** |
| --- | --- | --- | --- | --- | --- | --- | --- |
|  | **Min** | **Max** | **Av.** | **Min** | **Max** | **Av.** |  |
| Berlin | 0.04 | 0.34 |  | 0.04 | 0.60 |  | (Harrison et al., 2005; Schmidt-Bäumler et al., 1999) |
| Antarctic lakes | < LOD | 0.428 | 0.087 | 0.011 | 0.070 | 0.034 | (Harrison et al., 2005; Vanni et al., 2001) |
| Elsnig, ground water, upper level |  |  |  |  |  | 15 | (Harrison et al., 2005; Wennrich et al., 1995) |
| Ibid, lower level |  |  |  |  |  | 88 |  |

**Table S7:** Exposure effects achieved in years in the two lung cell lines following 24 and 48 hours of exposure to the value corresponding to the IC-50 values of each nitrophenol and their equimolar mixture.

| **IC-50 Values Exposure Concentration** | | **BEAS-2B** | | **A549** | |
| --- | --- | --- | --- | --- | --- |
|  |  | **24 Hours** | **48 Hours** | **24 Hours** | **48 Hours** |
| **2NP** | Cell Exposure Concentration | 397 µg mL^-1^  124 µg cm^-2^ | 31927 µg mL^-1^  125 µg cm^-2^ | 1.8 x 10^8^ µg mL^-1^  5.6 x 10^7^ µg cm^-2^ | 6.3 x 10^8^ µg mL^-1^  2.0 x 10^8^ µg cm^-2^ |
|  | Exposure effect Time | ~ 452 years | ~ 3600 years | ~ 2.0 x 10^8^ years | ~ 7.3 x 10^8^ years |
| **3NP** | Cell Exposure Concentration | 123 µg mL^-1^  40 µg cm^-2^ | 40 µg mL^-1^  13 µg cm^-2^ | 2505 µg mL^-1^  814 µg cm^-2^ | 109 µg mL^-1^  35 µg cm^-2^ |
|  | Exposure effect Time | ~ 146 years | ~ 47 years | ~ 2900 years | ~ 128 years |
| **4NP** | Cell Exposure Concentration | 93 µg mL^-1^  30 µg cm^-2^ | 43 µg mL^-1^  13 µg cm^-2^ | 3.5 x 10^5^ µg mL^-1^  ~1.1 x 10^4^ µg cm^-2^ | 552 µg mL^-1^  170 µg cm^-2^ |
|  | Exposure effect Time | ~ 110 years | ~ 51 years | ~ 41,000 years | ~ 655 years |
| **NP Mixtures** | Cell Exposure Concentration | 107 µg mL^-1^  35 µg cm^-2^ | 78 µg mL^-1^  25 µg cm^-2^ | 223 µg mL^-1^  72 µg cm^-2^ | 122 µg mL^-1^  40 µg cm^-2^ |
|  | Exposure effect Time | ~ 127 years | ~ 93 years | ~ 265 years | ~ 145 years |


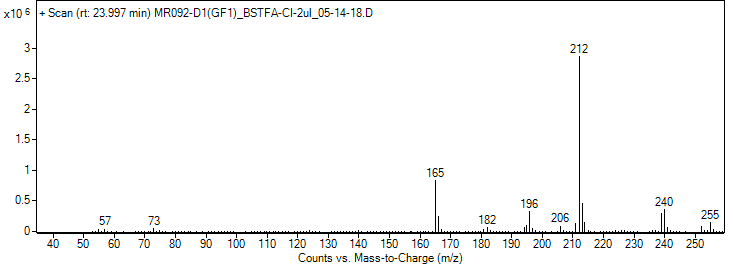


**4-Nitrophenol-TMS spectrum (EI mode): standard**


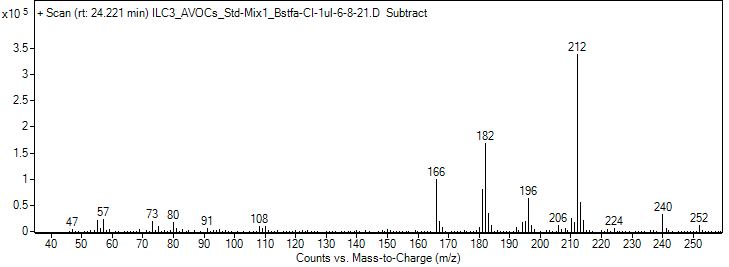

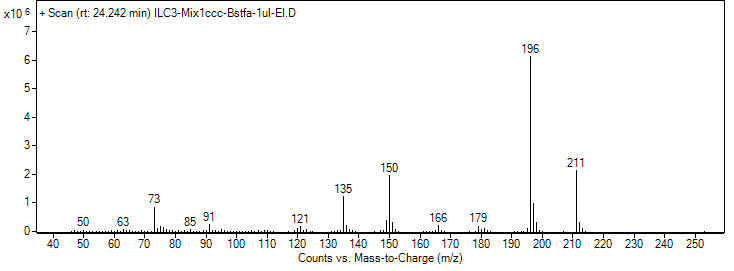


**4-Nitrophenol-TMS spectrum (CI mode): toluene SOA**

**4-Nitrophenol-TMS spectrum (CI mode): standard**

**(a)**

**(b)**

**(c)**

**Figure S1.** Mass spectra in (a) EI (70 EV, top panel) and (b) CI (middle panel) mode acquired for 4NP standard as trimethylsilyl (TMS) derivative. (c) MR092 smog chamber-generated SOA containing 4NP is shown in the bottom panel.


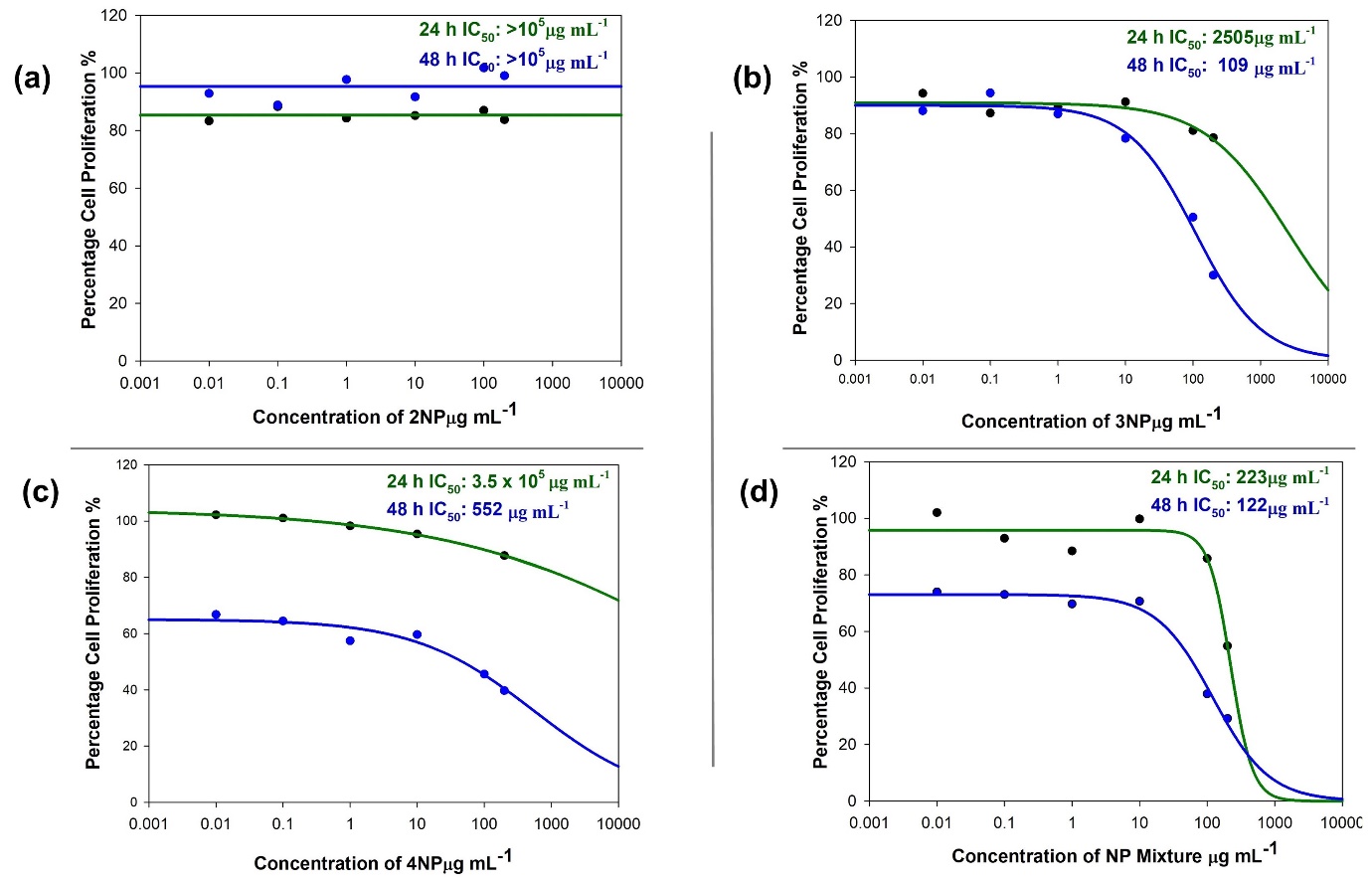


**Figure S2**. The dose-response curve of A549 cells treated with increasing concentration of NPs, as calculated through the MTT assay. The graphs show the inhibition curve generated at 24 h and 48 h of (a) the 2NP (b) the 3NP (c) the 4NP and (d) the NP mixture exposure. Individual IC50 values are also provided with each graph. Note similar dose response curves for A549 cells are provided in Figure S2 in SI.


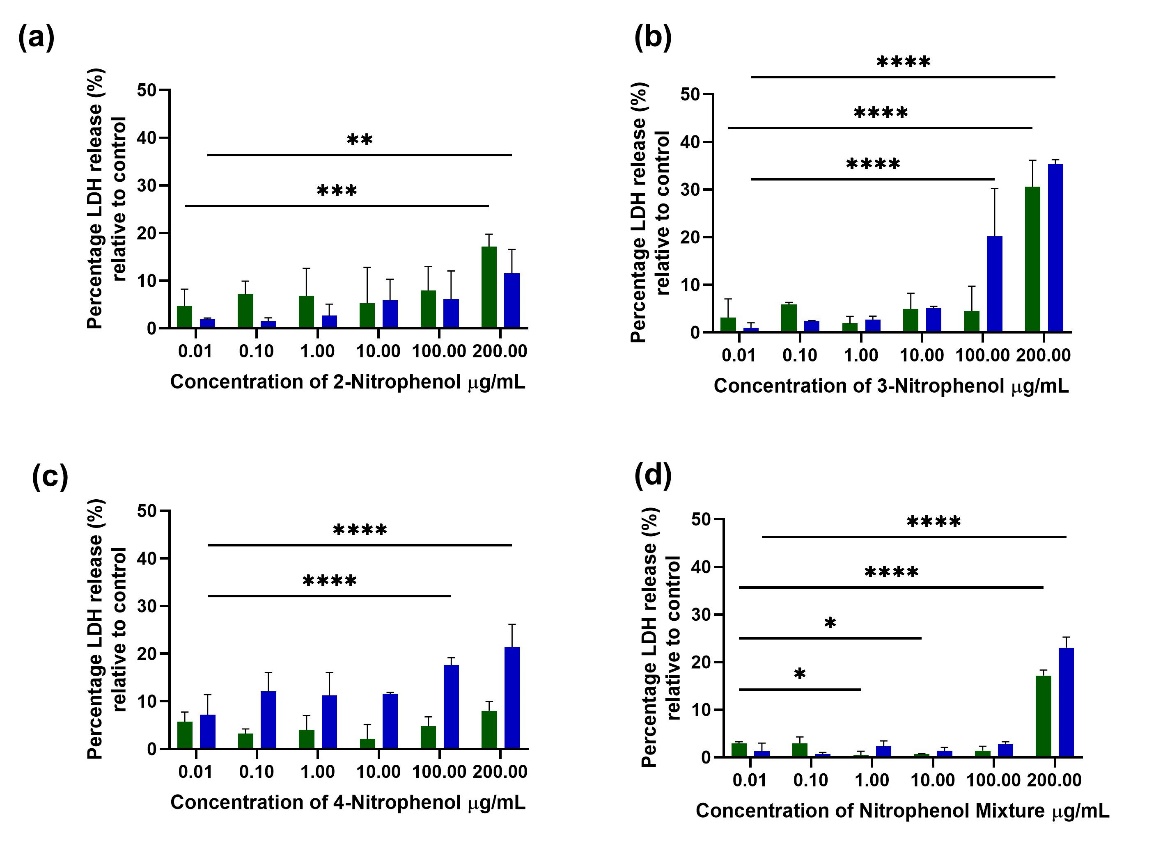


**Figure S3.** The difference in the percentage of LDH release when compared with untreated (control) A549 cells following 24 and 48 h exposure to (a) 2NP (b) 3NP (c) 4NP and (d) NP mixture. Results were statistically analyzed through a two-way Anova, followed by Sidak’s multiple comparison test. The p-value of <0.5 was considered statistically significant where **** corresponds to a p-value <0.0001


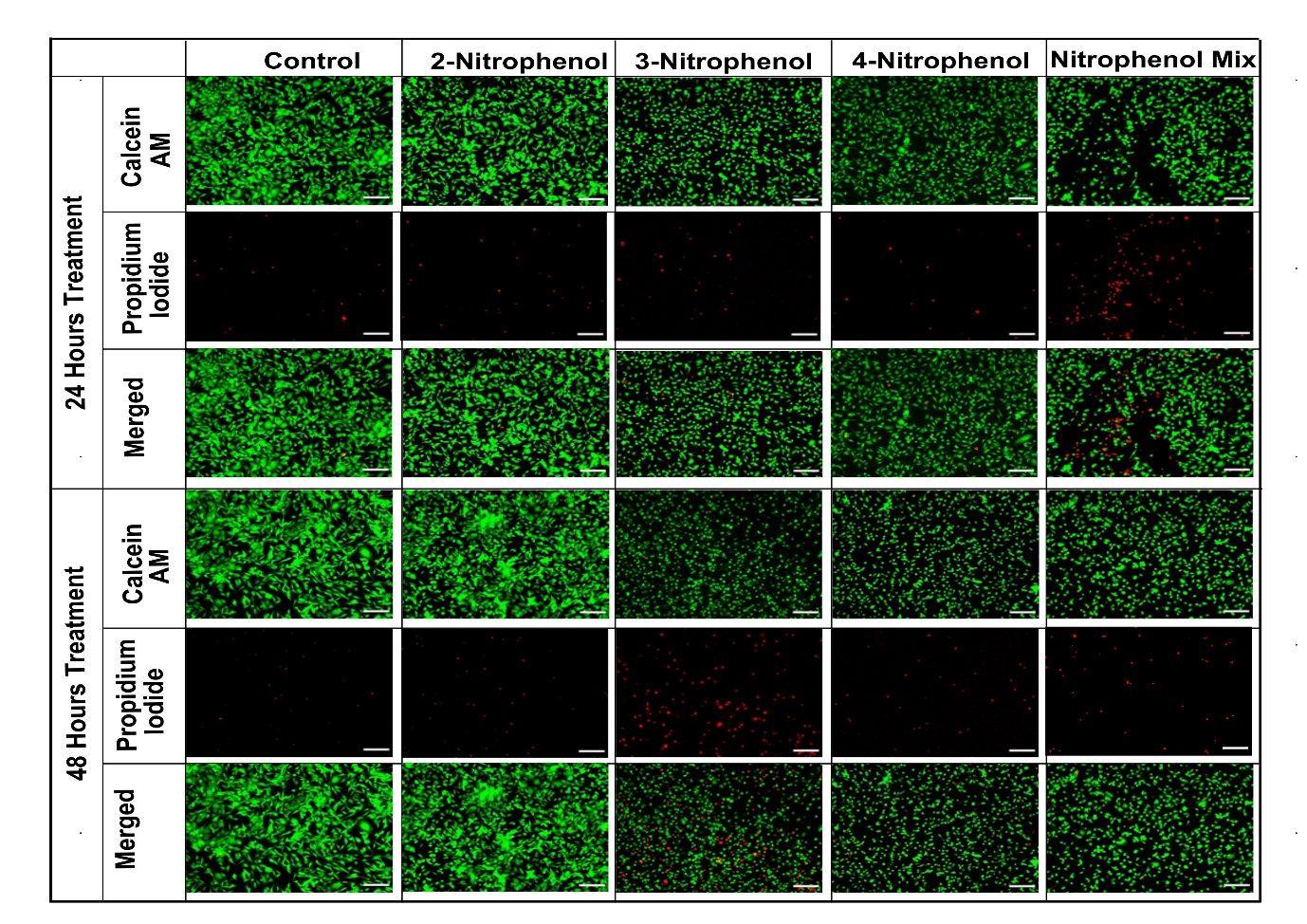


**Figure S4:** NP-treated BEAS-2B cells were visualized through calcein-AM/ propidium iodide (live/dead) staining using fluorescence microscopy. The calcein-AM (green) stained the live cells while propidium iodide (red) stained the dead cells and the merged channel shows the overlay of two images. The micrographs are scaled to a size of 50 µm. (a) The live/dead staining of BEAS-2B cells revealed the number of dead cells increased with increasing time of treatment; the highest number of cells were dead following exposure to the equimolar mixture of the NPs (i.e., 2NP, 3NP, and 4NP).


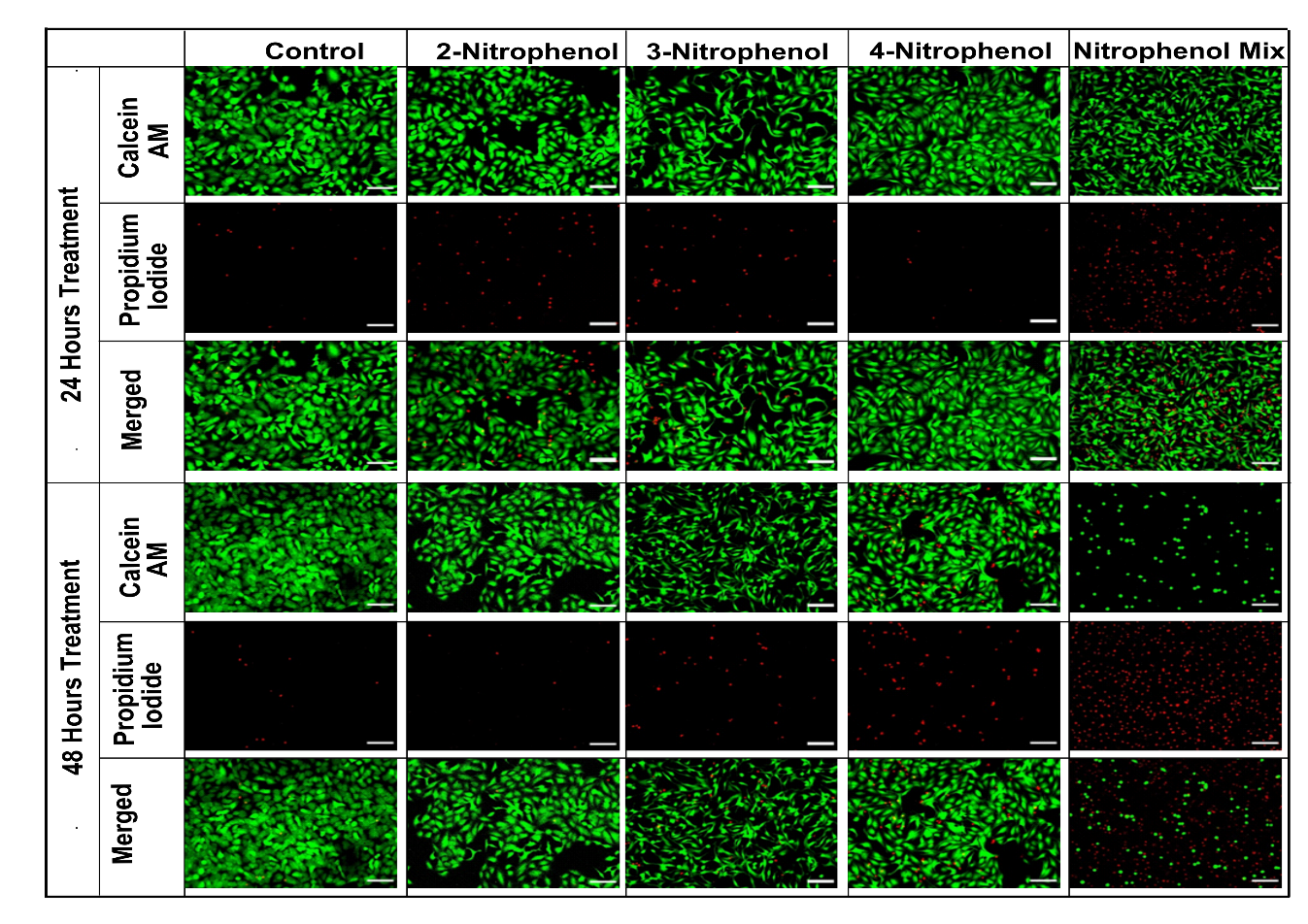


**Figure S5:** NP-treated A549 cells were visualized through live/dead staining using fluorescence microscopy The live/dead staining of A549 cells show similar results as determined by the LDH assay (i.e., negligible cells were dead after 24 and 48 hours of exposure to 2NP, while highest number of cells were dead following the treatment with equimolar NP mixtures).


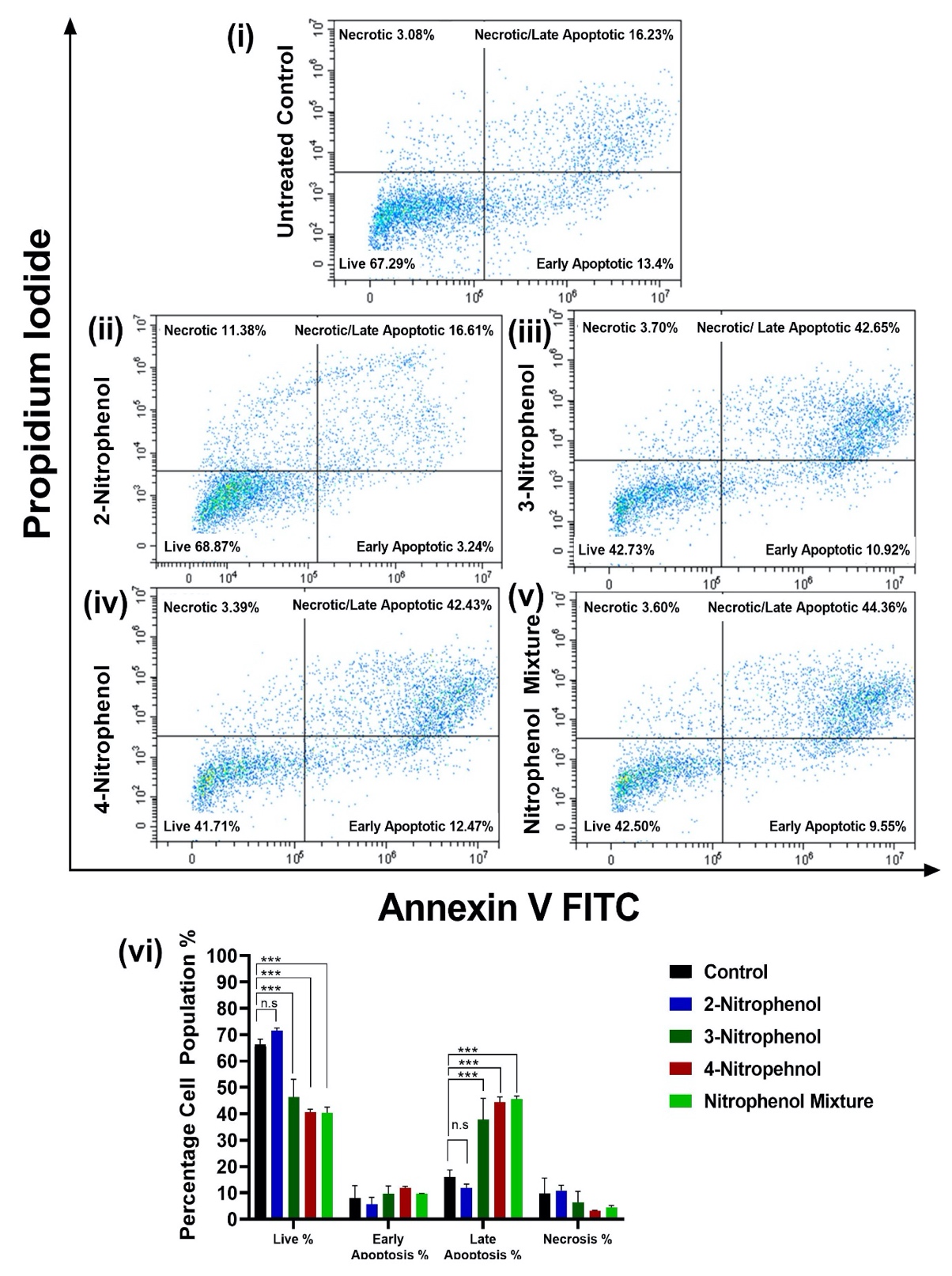


**Figure S6.** The percentages of BEAS-2B cell populations that died by various mechanisms (early apoptosis, late apoptosis/necrosis, and necrosis) or remained live after 200µg mL^-1^ NPs exposure for i) untreated control (ii) 2NP (iii) 3NP (iv) 4NP and (v) the NP equimolar mixture, determined using the Annexin-V/Propidium Iodide labeling and flow cytometry. Representative dot plot regards exposure at 48 h. One-way ANOVA with Dunnett’s multiple comparisons test was used to determine the statistical significance between treatment and controls groups. The p-value <0.05 was considered statistically significant for our analysis where *** indicates a p-value ≤0.001for our analysis where *** indicates a p-value ≤0.001

**Figure S7.** The percentages of A549 cell populations that died by various mechanisms (early apoptosis, late apoptosis/necrosis, and necrosis) or remained live after 200µg mL^-1^ NPs exposure for i) untreated control (ii) 2NP (iii) 3NP (iv) 4NP and (v) the NP equimolar mixture, determined using the Annexin-V/Propidium Iodide labeling and flow cytometry. Representative dot plot regards exposure at 24 h. One-way ANOVA with Dunnett’s multiple comparisons test was used to determine the statistical significance between treatment and controls groups. The p-value <0.05 was considered statistically significant for our analysis where *** indicates a p-value ≤0.001for our analysis where *** indicates a p-value ≤0.001


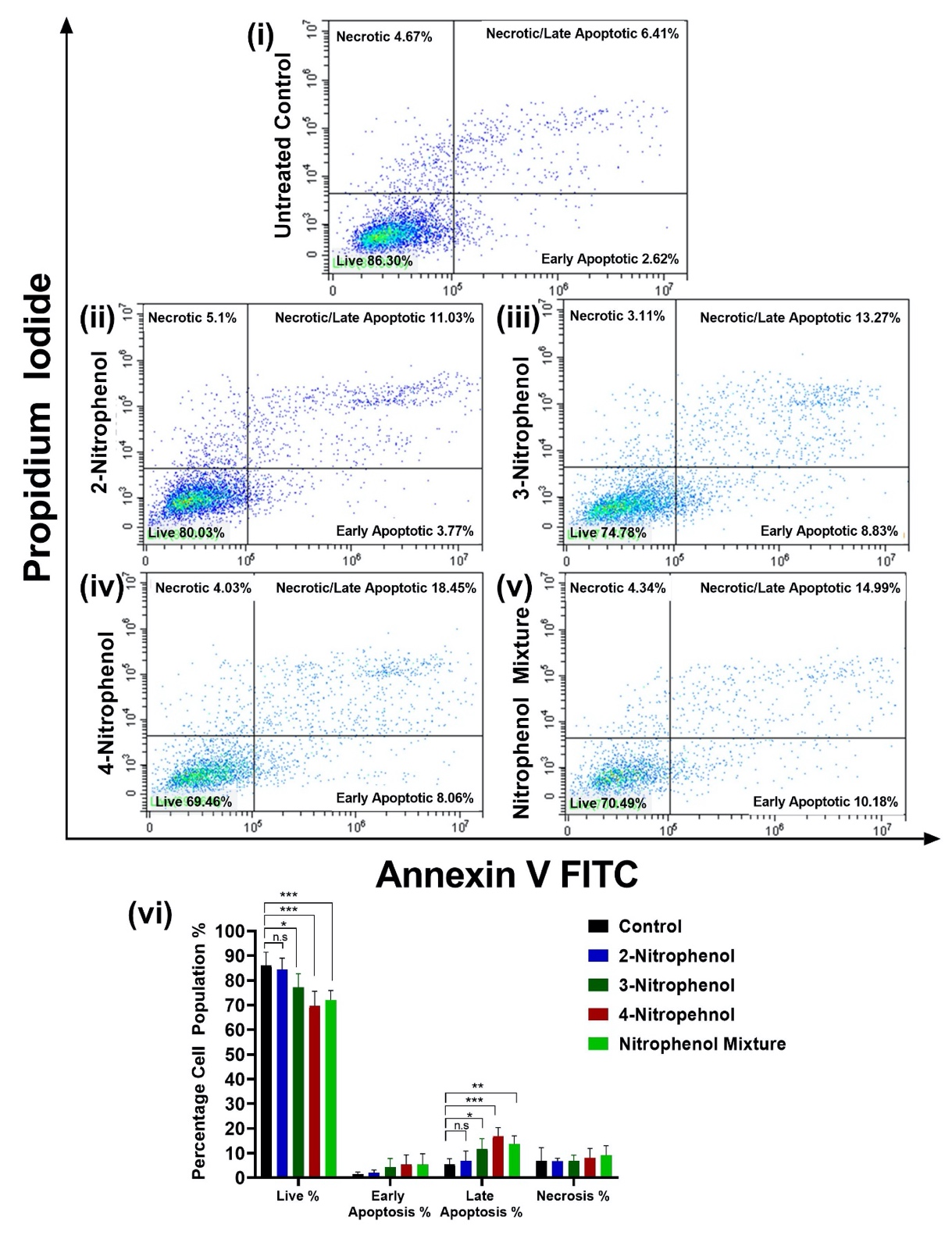

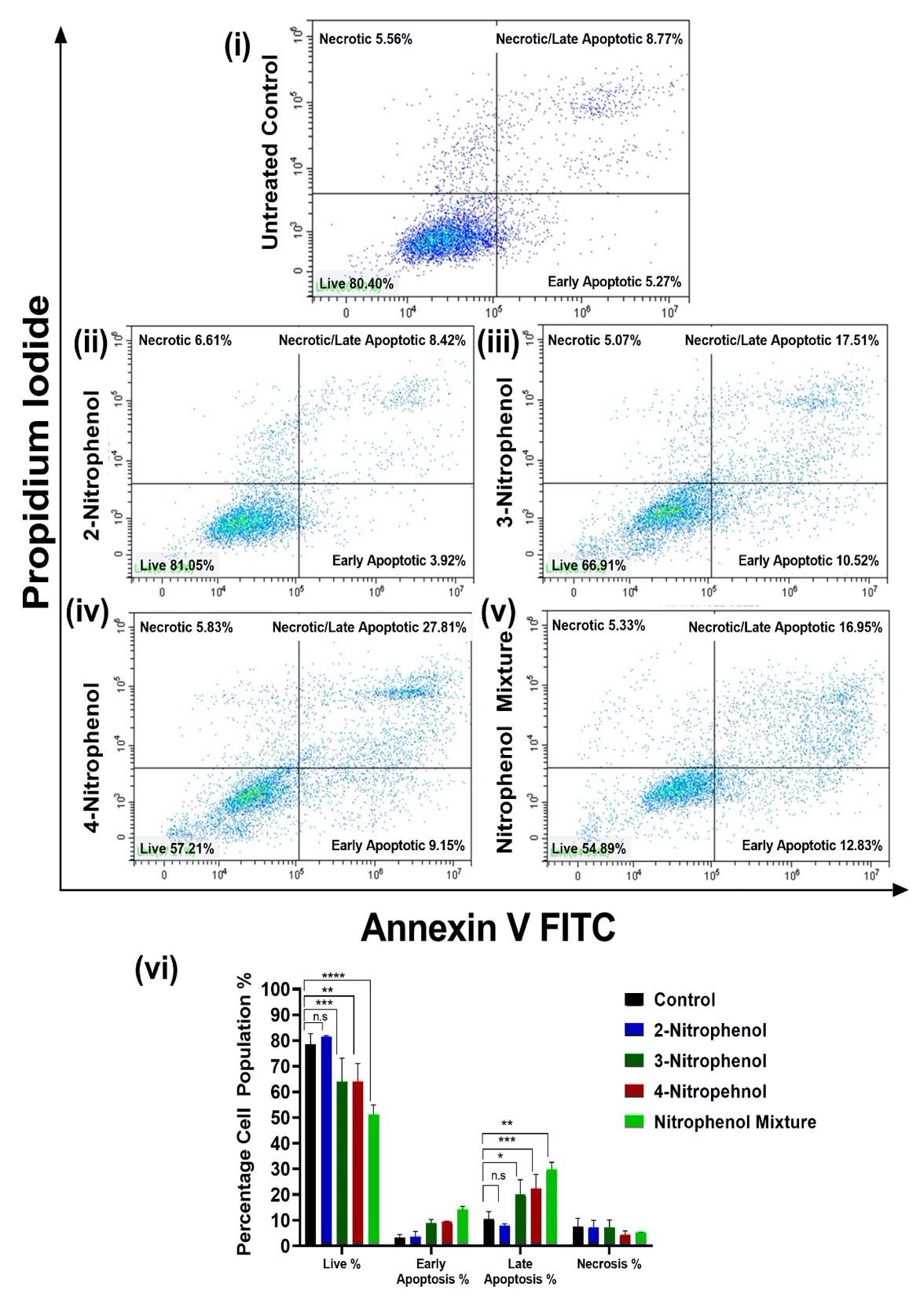


**Figure S8.** The percentages of A549 cell populations that died by various mechanisms (early apoptosis, late apoptosis/necrosis, and necrosis) or remained live after 200µg mL-1 NPs exposure for i) untreated control (ii) 2NP (iii) 3NP (iv) 4NP and (v) the NP equimolar mixture, determined using the Annexin-V/Propidium Iodide labeling and flow cytometry. Representative dot plot regards exposure at 48 h. One-way ANOVA with Dunnett’s multiple comparisons test was used to determine the statistical significance between treatment and controls groups. The p-value <0.05 was considered statistically significant for our analysis where *** indicates a p-value ≤0.001for our analysis where *** indicates a p-value ≤0.001


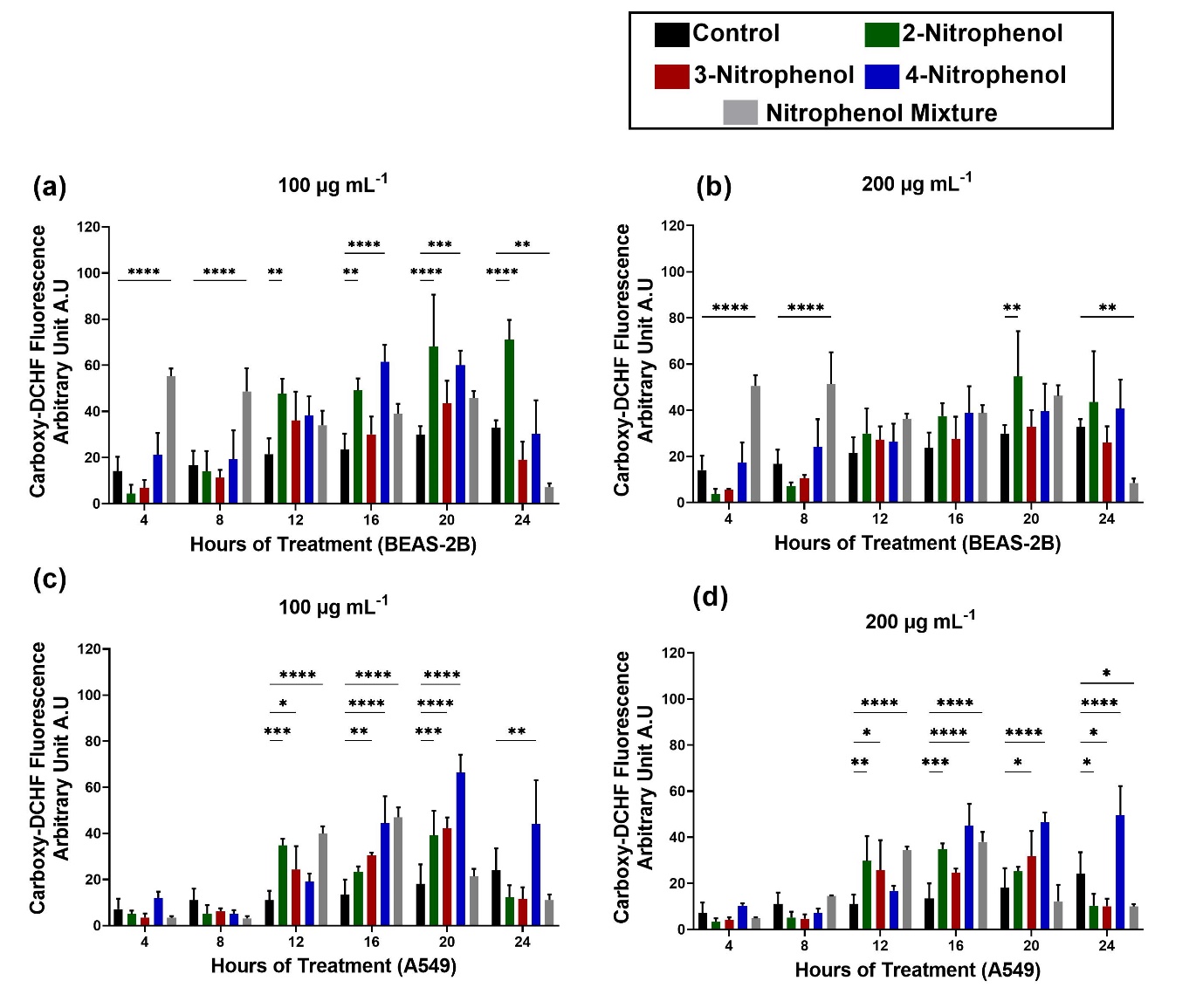


**Figure S9** (a) The change in carboxy-H_2_DCHF signal was determined using spectrophotometer plate-reader after every four hours up to 24 hours post exposure using the concentration of NPs as: (a) 100 µg mL^-1^ in BEAS-2B; (b) 200 µg mL^-1^ in BEAS-2B; (c) 100 µg mL^-1^ in A549; and (d) 200 µg mL^-1^ in A549 of the 2NP, 3NP, and 4NP as well as their equimolar mixture. The increase in the carboxy-H_2_DCHF signal relative to the untreated control was analyzed statistically through two-way Anova followed by Dunnett’s multiple comparison test. The p-value <0.05 was considered statistically significant for our analysis where **** indicates a p-value ≤0.0001.


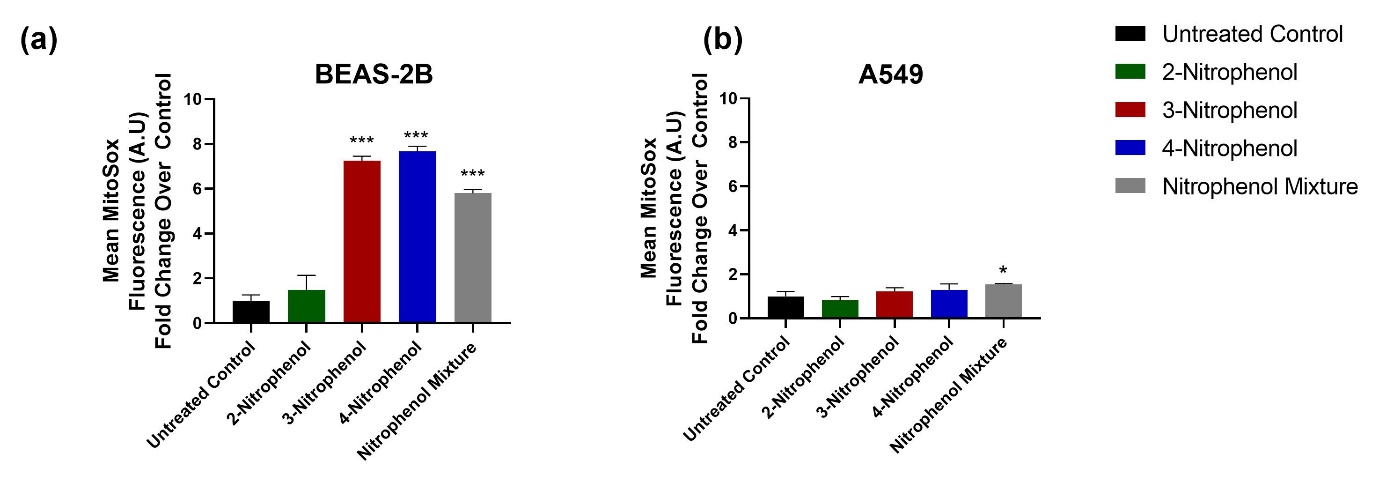


**Figure S10:** The change in MitoSox signal was further analyzed through flow cytometry. MitoSox fluorescence was observed as a function of change in mitochondrial superoxide production following treatment with 200 µg mL^-1^ of the 2NP, 3NP, and 4NP as well as their equimolar mixture after 8 hours of exposure in: (a) BEAS-2B cell lines; and (b) A549 cell lines. One-way Anova followed by Dunnett’s multiple comparison test was run on the untreated control versus treatment group to analyze the significant difference in the fold change of fluorescence signal where the p-value <0.05 was considered statistically significant for our analysis and *** indicates a p-value ≤0.001.


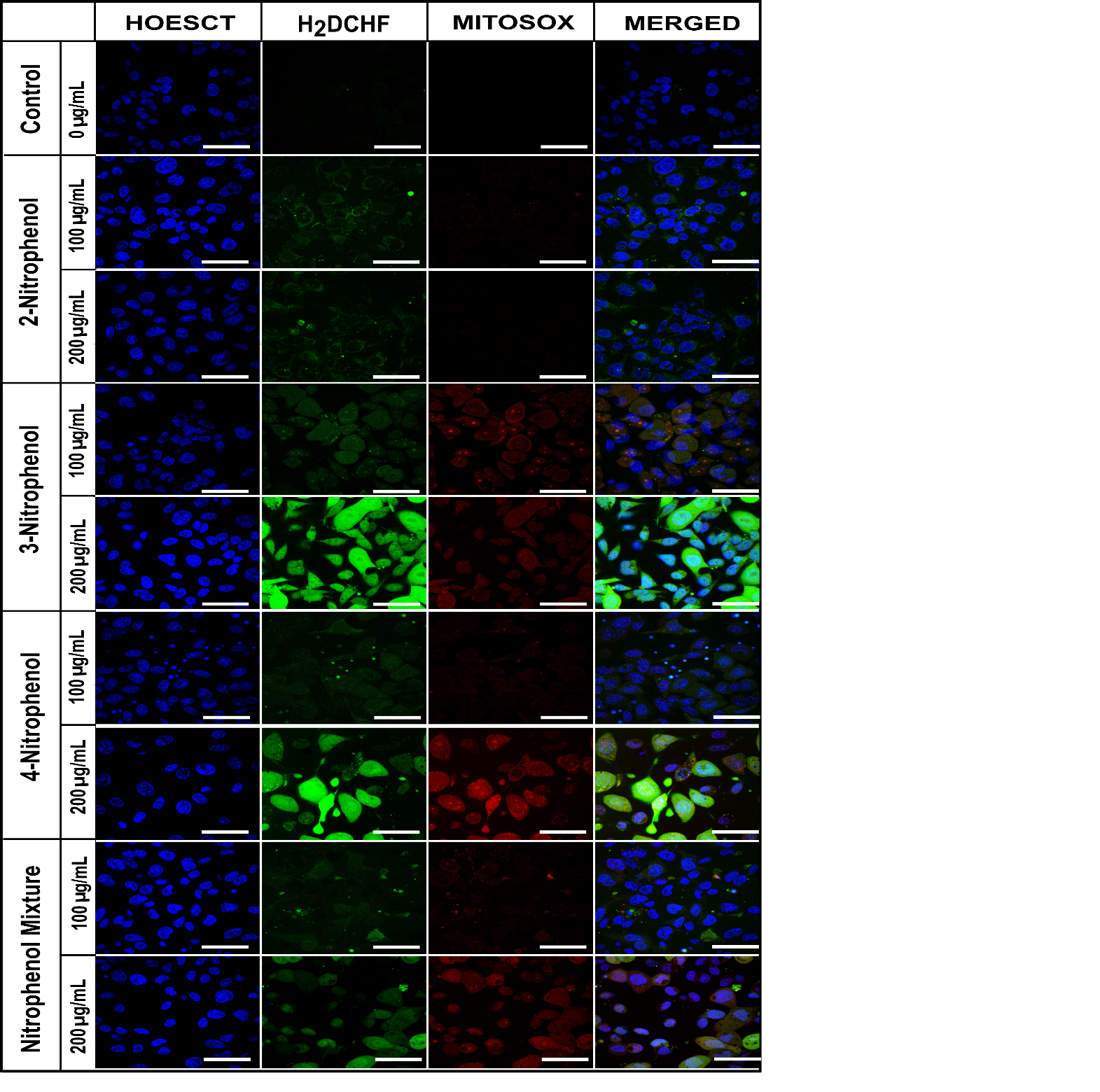


**Figure S11.** Confocal microscopy micrographs showing the OS signals following the NP treatments (100 and 200 µg mL^-1^) in A549 cells. Blue color shows the nuclei of the live cells, green – the general ROS buildup, and red – the mitochondrial superoxide. The merged channel overlays all colors. Scale bars show 25 µm size


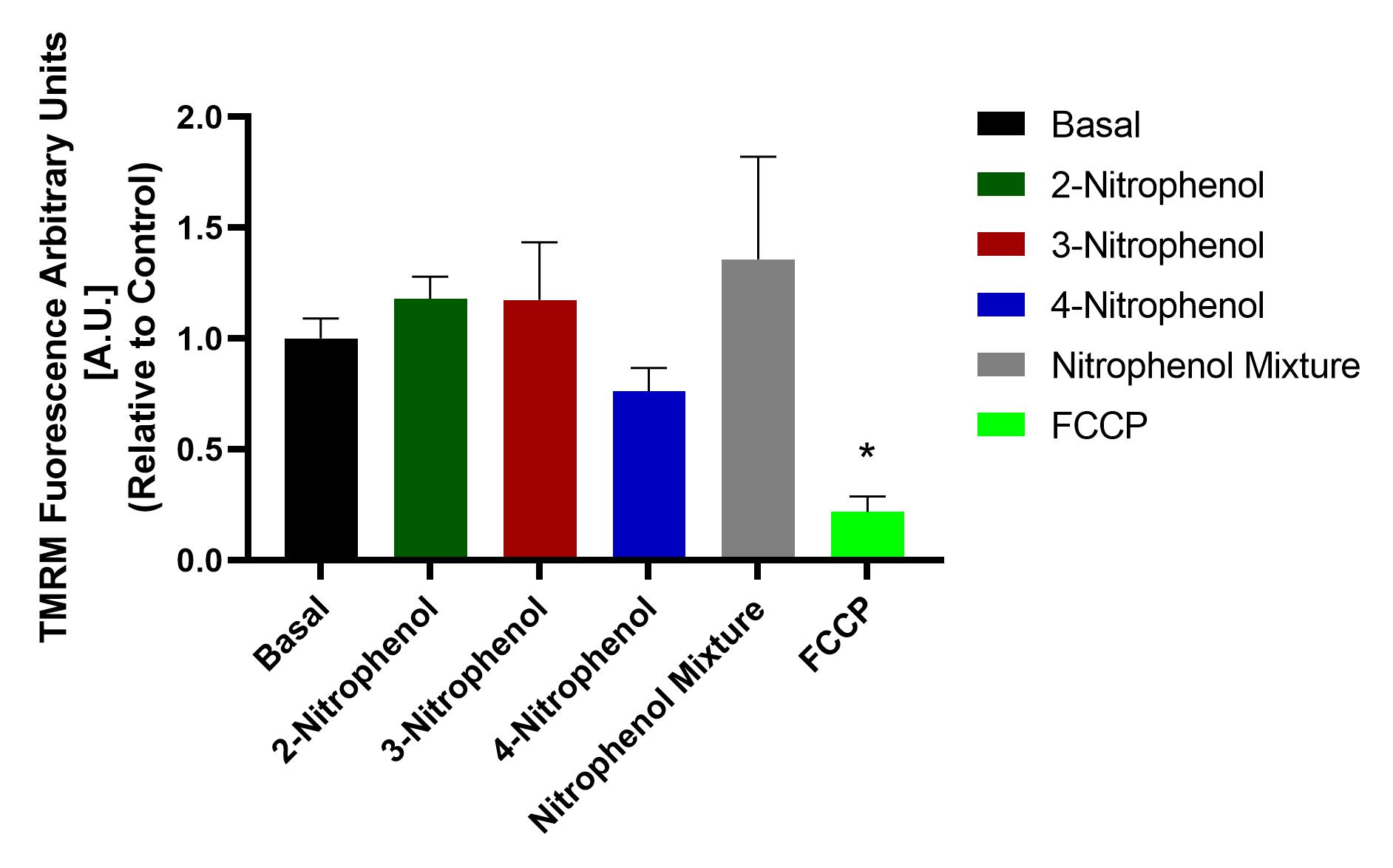


**Figure S12.** TMRM measurement of the membrane potential in A549 cells treated for 24h with the 200-µg mL^-1^ of 2NP, 3NP, 4NP, and their equimolar mixture. Treatments with carbonyl cyanide 4-(trifluoromethoxy) phenylhydrazone (FCCP) were a positive control for ΔΨm decrease. ANOVA probabilities of incorrectly concluding that differences occurred were: p<0.05 and **p≤0.01.

**References**

Al-Naiema, I.M., Stone, E.A., 2017. Evaluation of anthropogenic secondary organic aerosol tracers from aromatic hydrocarbons. Atmos. Chem. Phys. 17, 2053-2065. 10.5194/acp-17-2053-2017.

Asman, W.A.H., Jørgensen, A., Bossi, R., Vejrup, K.V., Bügel Mogensen, B., Glasius, M., 2005. Wet deposition of pesticides and nitrophenols at two sites in Denmark: measurements and contributions from regional sources. Chemosphere 59, 1023-1031. <https://doi.org/10.1016/j.chemosphere.2004.11.048>.

Belloli, R., Barletta, B., Bolzacchini, E., Meinardi, S., Orlandi, M., Rindone, B., 1999. Determination of toxic nitrophenols in the atmosphere by high-performance liquid chromatography. Journal of Chromatography A 846, 277-281. <https://doi.org/10.1016/S0021-9673(99)00030-8>.

Belloli, R., Bolzacchini, E., Clerici, L., Rindone, B., Sesana, G., Librando, V., 2006. Nitrophenols in air and rainwater. Environ. Eng. Sci. 23, 405-415. 10.1089/ees.2006.23.405.

Cecinato, A., Di Palo, V., Pomata, D., Tomasi Scianò, M.C., Possanzini, M., 2005. Measurement of phase-distributed nitrophenols in Rome ambient air. Chemosphere 59, 679-683. <https://doi.org/10.1016/j.chemosphere.2004.10.045>.

Chow, K.S., Huang, X.H.H., Yu, J.Z., 2016. Quantification of nitroaromatic compounds in atmospheric fine particulate matter in Hong Kong over 3 years: field measurement evidence for secondary formation derived from biomass burning emissions. Environmental Chemistry 13, 665-673. <https://doi.org/10.1071/EN15174>.

Delhomme, O., Morville, S., Millet, M., 2010. Seasonal and diurnal variations of atmospheric concentrations of phenols and nitrophenols measured in the Strasbourg area, France. Atmos. Pollut. Res. 1, 16-22. 10.5094/APR.2010.003.

Harrison, M.A.J., Barra, S., Borghesi, D., Vione, D., Arsene, C., Iulian Olariu, R., 2005. Nitrated phenols in the atmosphere: a review. Atmospheric Environment 39, 231-248. <http://dx.doi.org/10.1016/j.atmosenv.2004.09.044>.

Irei, S., Stupak, J., Gong, X., Chan, T.-W., Cox, M., McLaren, R., Rudolph, J., 2017. Molecular marker study of particulate organic matter in Southern Ontario air. Journal of Analytical Methods in Chemistry 2017, 19. 10.1155/2017/3504274.

Khan, F., Kwapiszewska, K., Zhang, Y., Chen, Y., Lambe, A.T., Kołodziejczyk, A., Jalal, N., Rudzinski, K., Martínez-Romero, A., Fry, R.C., Surratt, J.D., Szmigielski, R., 2021. Toxicological Responses of α-Pinene-Derived Secondary Organic Aerosol and Its Molecular Tracers in Human Lung Cell Lines. Chemical Research in Toxicology 34, 817-832. 10.1021/acs.chemrestox.0c00409.

Kitanovski, Z., Grgic, I., Vermeylen, R., Claeys, M., Maenhaut, W., 2012. Liquid chromatography tandem mass spectrometry method for characterization of monoaromatic nitro-compounds in atmospheric particulate matter. J. Chromatogr. A 1268, 35-43. 10.1016/j.chroma.2012.10.021.

Kitanovski, Z., Hovorka, J., Kuta, J., Leoni, C., Prokeš, R., Sáňka, O., Shahpoury, P., Lammel, G., 2020a. Nitrated monoaromatic hydrocarbons (nitrophenols, nitrocatechols, nitrosalicylic acids) in ambient air: levels, mass size distributions and inhalation bioaccessibility. Environmental Science and Pollution Research. 10.1007/s11356-020-09540-3.

Kitanovski, Z., Shahpoury, P., Samara, C., Voliotis, A., Lammel, G., 2020b. Composition and mass size distribution of nitrated and oxygenated aromatic compounds in ambient particulate matter from southern and central Europe – implications for the origin. Atmos. Chem. Phys. 20, 2471-2487. 10.5194/acp-20-2471-2020.

Lanzafame, G.M., Srivastava, D., Favez, O., Bandowe, B.A.M., Shahpoury, P., Lammel, G., Bonnaire, N., Alleman, L.Y., Couvidat, F., Bessagnet, B., Albinet, A., 2021. One-year measurements of secondary organic aerosol (SOA) markers in the Paris region (France): Concentrations, gas/particle partitioning and SOA source apportionment. Science of The Total Environment 757, 143921. <https://doi.org/10.1016/j.scitotenv.2020.143921>.

Leuenberger, C., Czuczwa, J., Tremp, J., Giger, W., 1988. Nitrated phenols in rain: Atmospheric occurrence of phytotoxic pollutants. Chemosphere 17, 511-515. <https://doi.org/10.1016/0045-6535(88)90026-4>.

Leuenberger, C., Ligocki, M.P., Pankow, J.F., 1985. Trace organic compounds in rain. 4. Identities, concentrations, and scavenging mechanisms for phenols in urban air and rain. Environmental Science & Technology 19, 1053-1058. 10.1021/es00141a005.

Levsen, K., Behnert, S., Mußmann, P., Raabe, M., Prieß, B., 1993. Organic Compounds In Cloud And Rain Water. International Journal of Environmental Analytical Chemistry 52, 87-97. 10.1080/03067319308042851.

Levsen, K., Behnert, S., Prieß, B., Svoboda, M., Winkeler, H.-D., Zietlow, J., 1990. Organic compounds in precipitation. Chemosphere 21, 1037-1061. <https://doi.org/10.1016/0045-6535(90)90127-F>.

Li, X., Jiang, L., Hoa, L.P., Lyu, Y., Xu, T., Yang, X., Iinuma, Y., Chen, J., Herrmann, H., 2016. Size distribution of particle-phase sugar and nitrophenol tracers during severe urban haze episodes in Shanghai. Atmospheric Environment 145, 115-127. <https://doi.org/10.1016/j.atmosenv.2016.09.030>.

Lüttke, J., Levsen, K., Acker, K., Wieprecht, W., Möller, D., 1999. Phenols and Nitrated Phenols in Clouds at Mount Brocken. International Journal of Environmental Analytical Chemistry 74, 69-89. 10.1080/03067319908031417.

Mohr, C., Lopez-Hilfiker, F.D., Zotter, P., Prévôt, A.S.H., Xu, L., Ng, N.L., Herndon, S.C., Williams, L.R., Franklin, J.P., Zahniser, M.S., Worsnop, D.R., Knighton, W.B., Aiken, A.C., Gorkowski, K.J., Dubey, M.K., Allan, J.D., Thornton, J.A., 2013. Contribution of Nitrated Phenols to Wood Burning Brown Carbon Light Absorption in Detling, United Kingdom during Winter Time. Environmental Science & Technology 47, 6316-6324. 10.1021/es400683v.

Nojima, K., Kawaguchi, A., Ohya, T., Kanno, S., Hirobe, M., 1983. Studies on Photochemical Reaction of Air Pollutants. X. Identification of Nitrophenols in Suspended Particulates. CHEMICAL & PHARMACEUTICAL BULLETIN 31, 1047-1051. 10.1248/cpb.31.1047.

Özel, M.Z., Hamilton, J.F., Lewis, A.C., 2011. New sensitive and quantitative analysis method for organic nitrogen compounds in urban aerosol samples. Environmental Science & Technology 45, 1497-1505. 10.1021/es102528g.

Richartz, H., Reischl, A., Trautner, F., Hutzinger, O., 1990. Nitrated phenols in fog. Atmospheric Environment. Part A. General Topics 24, 3067-3071. <https://doi.org/10.1016/0960-1686(90)90485-6>.

Rippen, G., Zietz, E., Frank, R., Knacker, T., Klöpffer, W., 1987. Do airborne nitrophenols contribute to forest decline? Environmental Technology Letters 8, 475-482. 10.1080/09593338709384508.

Rubio, M.A., Lissi, E., Herrera, N., Pérez, V., Fuentes, N., 2012. Phenol and nitrophenols in the air and dew waters of Santiago de Chile. Chemosphere 86, 1035-1039. <https://doi.org/10.1016/j.chemosphere.2011.11.046>.

Schmidt-Bäumler, K., Heberer, T., Stan, H.-J., 1999. Occurrence and distribution of organic contaminants in the aquatic system in Berlin.Part II: Substituted phenols in Berlin surface water. Acta hydrochimica et hydrobiologica 27, 143-149. 10.1002/(sici)1521-401x(199905)27:3<143::Aid-aheh143>3.0.Co;2-9.

Schulz, C., Farkas, L., Wolf, K., Krأ╡Tzel, K., Eissner, G., Pfeifer, M., 2002. Differences in LPS-Induced Activation of Bronchial Epithelial Cells (BEAS-2B) and Type II-Like Pneumocytes (A-549). Scandinavian Journal of Immunology 56, 294-302. <https://doi.org/10.1046/j.1365-3083.2002.01137.x>.

Teich, M., Pinxteren, D., Herrmann, H., 2014. Determination of nitrophenolic compounds from atmospheric particles using hollow-fiber liquid-phase microextraction and capillary electrophoresis/mass spectrometry analysis. Electrophoresis 35, 1353-1361. 10.1002/elps.201300448.

Vanni, A., Pellegrino, V., Gamberini, R., Calabria, A., 2001. An Evidence for Nitrophenols Contamination in Antarctic Fresh-Water and Snow. Simultaneous Determination of Nitrophenols and Nitroarenes at ng/L Levels. International Journal of Environmental Analytical Chemistry 79, 349-365. 10.1080/03067310108044394.

Wennrich, L., Efer, J., Engewald, W., 1995. Gas chromatographic trace analysis of underivatized nitrophenols. Chromatographia 41, 361-366. 10.1007/BF02688052.

Yassine, M.M., Suski, M., Dabek-Zlotorzynska, E., 2020. Characterization of benzene polycarboxylic acids and polar nitroaromatic compounds in atmospheric aerosols using UPLC-MS/MS. Journal of Chromatography A 1630, 461507. <https://doi.org/10.1016/j.chroma.2020.461507>.

Zhang, Y.Y., Müller, L., Winterhalter, R., Moortgat, G.K., Hoffmann, T., Pöschl, U., 2010. Seasonal cycle and temperature dependence of pinene oxidation products, dicarboxylic acids and nitrophenols in fine and coarse air particulate matter. Atmos. Chem. Phys. 10, 7859-7873. 10.5194/acp-10-7859-2010.
